# Cholesterol homeostasis and lipid raft dynamics at the basis of tumor-induced immune dysfunction in chronic lymphocytic leukemia

**DOI:** 10.1038/s41423-025-01262-1

**Published:** 2025-03-04

**Authors:** Chaja F. Jacobs, Fleur S. Peters, Elena Camerini, Gaspard Cretenet, Joanne Rietveld, Bauke V. Schomakers, Michel van Weeghel, Nico Hahn, Sanne G. S. Verberk, Jan Van den Bossche, Mirjam Langeveld, Fleur Kleijwegt, Eric Eldering, Noam Zelcer, Arnon P. Kater, Helga Simon-Molas

**Affiliations:** 1 Department of Experimental Immunology, Amsterdam University Medical Centers, University of Amsterdam, Amsterdam, The Netherlands; 2 Department of Hematology, Amsterdam University Medical Centers, University of Amsterdam, Amsterdam, The Netherlands; 3 Amsterdam Institute for Immunology and Infectious Diseases, Amsterdam, The Netherlands; 4 Cancer Center Amsterdam, Cancer Immunology Program, Amsterdam, The Netherlands; 5 Laboratory Genetic Metabolic Diseases, Amsterdam University Medical Centers, University of Amsterdam, Amsterdam, The Netherlands; 6 Core Facility Metabolomics, Amsterdam University Medical Centers, Amsterdam, The Netherlands; 7 Amsterdam Cardiovascular Sciences (ACS), Amsterdam University Medical Centers, Amsterdam, Amsterdam, The Netherlands; 8 Amsterdam Gastroenterology Endocrinology Metabolism (AGEM), Amsterdam University Medical Centers, Amsterdam, The Netherlands; 9 Department of Molecular Cell Biology and Immunology, Amsterdam University Medical Centers, Amsterdam, The Netherlands; 10 Department of Endocrinology and Metabolism, Amsterdam University Medical Centers, University of Amsterdam, Amsterdam, The Netherlands; 11 Rode Kruis Hospital, Beverwijk, The Netherlands; 12 Lymphoma and Myeloma Center Amsterdam, Amsterdam, The Netherlands; 13 Department of Medical Biochemistry, Amsterdam University Medical Centers, University of Amsterdam, Amsterdam, The Netherlands

**Keywords:** Leukemia, Lipid metabolism, T-cell, Immunotherapy, Cholesterol, Immunosurveillance, Chronic lymphocytic leukaemia, T cells, Cancer metabolism

## Abstract

Autologous T-cell therapies show limited efficacy in chronic lymphocytic leukemia (CLL), where acquired immune dysfunction prevails. In CLL, disturbed mitochondrial metabolism has been linked to defective T-cell activation and proliferation. Recent research suggests that lipid metabolism regulates mitochondrial function and differentiation in T cells, yet its role in CLL remains unexplored. This comprehensive study compares T-cell lipid metabolism in CLL patients and healthy donors, revealing critical dependence on exogenous cholesterol for human T-cell expansion following TCR-mediated activation. Using multi-omics and functional assays, we found that T cells present in viably frozen samples of patients with CLL (CLL T cells) showed impaired adaptation to cholesterol deprivation and inadequate upregulation of key lipid metabolism transcription factors. CLL T cells exhibited altered lipid storage, with increased triacylglycerols and decreased cholesterol, and inefficient fatty acid oxidation (FAO). Functional consequences of reduced FAO in T cells were studied using samples from patients with inherent FAO disorders. Reduced FAO was associated with lower T-cell activation but did not affect proliferation. This implicates low cholesterol levels as a primary factor limiting T-cell proliferation in CLL. CLL T cells displayed fewer and less clustered lipid rafts, potentially explaining the impaired immune synapse formation observed in these patients. Our findings highlight significant disruptions in lipid metabolism as drivers of functional deficiencies in CLL T cells, underscoring the pivotal role of cholesterol in T-cell proliferation. This study suggests that modulating cholesterol metabolism could enhance T-cell function in CLL, presenting novel immunotherapeutic approaches to improve outcome in this challenging disease.

## Introduction

Chronic lymphocytic leukemia (CLL) is the most abundant leukemia in the Caucasian population and is characterized by a progressive expansion of clonal mature B cells, mainly in blood, lymphoid tissues, and bone marrow. In the last decade, the treatment landscape for CLL was revolutionized due to the establishment of both the critical role of the B-cell receptor (BCR) signaling pathway as well as the dependency of CLL cells on the anti-apoptotic protein Bcl-2. Targeted agents against Bruton’s tyrosine kinase, a key kinase downstream of the BCR, and against Bcl-2 are now the preferred first- and/or second-line treatments in CLL patients [1]. Despite their success, these drugs are not curative, which is reflected by the emergence of BTK-inhibitor and BCL-2 inhibitor (double class-) resistance in patients, a condition associated with very poor outcome [2]. Hence, an unmet medical need exists for additional therapeutic strategies with curative potential.

Autologous T-cell-based therapies, such as CD19-directed chimeric antigen receptor (CAR) T cells, demonstrate durable anti-tumor responses in several aggressive hematological malignancies [3, 4]. However, only a small minority of CLL patients experience enduring responses from these therapies [5]. This lack of efficacy has been ascribed to acquired T-cell dysfunction that is often observed in CLL patients; a feature which likely develops through interactions between tumor cells and T cells [6–8]. Compared to T cells from age-matched healthy donors (HD), T cells from CLL patients are skewed towards effector subtypes, have an inverted CD4:CD8 ratio and exhibit reduced immune synapse (IS) formation, activation, proliferation and degranulation potential upon T-cell receptor (TCR) ligation [9–13]. However, the underlying mechanism(s) behind this CLL-imposed T-cell dysfunction is largely unclear.

Evidence from the solid tumor field shows that cancer-related T-cell dysfunction is associated with metabolic defects [14, 15]. Rapid shifts in metabolic processes underlie proper T-cell responses, determining the function and phenotype of T cells (extensively reviewed by Buck et al.) [16]. Upon TCR engagement, an early glycolytic switch is followed by an increase in mitochondrial activity. These processes demand differential fueling by glucose, amino acids, and lipids [16, 17]. Besides this classical view of the involvement of metabolism in T cell activation, metabolites—lipids in particular—have been recently shown to propagate and amplify the signal from the TCR by being an essential part of the IS for example, as reviewed by Wilfahrt et al. [18].

Whilst the contribution of glucose and glutamine upon TCR engagement has been well defined [19, 20] and their requirement for proper tumor immune surveillance has been studied [21, 22], the role of lipids in T cell homeostasis has only recently received attention. Lipids can be categorized into several classes, including fatty acids (FA), glycerolipids, and sterols, among others. In T cells, free FA are used for energy production in mitochondria and are essential for the synthesis of glycerophospholipids, the main components of cellular membranes. Glycerolipids consist of a glycerol molecule with one, two, or three FA (mono-, di, or triacylglycerols (TAG)). TAG and sterols constitute the so-called neutral lipids and can be stored in the cytoplasm for later use for energy production or for the building of new membranes [23]. Fluctuations in the amount and type of available lipids influence T-cell subset differentiation, proliferation, and survival [23, 24], with most literature focusing on the role of mitochondrial FA oxidation (FAO) in the development of memory and regulatory T cells [25, 26]. We have recently demonstrated that etomoxir, an inhibitor of FAO, diminished OXPHOS activity in malignant CLL cells, indicating these cells use lipids to fuel part of their metabolic demands [27]. Besides, a connection between poor CLL prognosis and high expression of lipoprotein lipase (LPL), an enzyme typically found on the surface of endothelial cells and macrophages involved in hydrolyzing lipoprotein-associated TAG, was identified previously [28]. Intriguingly, this link was not attributed to increased LPL activity, as it has been observed that LPL is predominantly inactive in CLL [29]. Rather, it seems that increased LPL expression is part of a transcriptional program that leads to elevated expression of genes related to lipid utilization [29], and a recent study has defined a FA metabolism-related expression signature to have predictive value for CLL prognosis [30]. Consequently, competition for lipid resources between malignant CLL cells and T cells could contribute to the acquired T-cell dysfunction in this disease.

We and others recently found that T cells present in viably frozen samples of patients with confirmed CLL (CLL T cells) have impaired mitochondrial plasticity and defective mitochondrial function alongside decreased activation and proliferation capacity [8, 11, 31, 32]. Given the crucial role of lipids in these processes, we investigated whether lipid metabolism differs in T cells from CLL patients (CLL T cells) compared to HD, and if there is a mechanistic link between altered lipid metabolism and acquired T-cell dysfunction, particularly reduced proliferation. By a comprehensive, in-depth characterization of key branches of T-cell lipid metabolism, we found that CLL T cells are highly dependent on exogenous cholesterol for proliferation and fail to increase lipid uptake, synthesis, and utilization within the first 2 days upon TCR engagement, as healthy T cells do. In HD T cells, this response is regulated through a combined increase in sterol regulatory element-binding proteins (SREBPs) and peroxisome proliferator-activated receptors (PPARs) transcriptional programs. T cells from CLL patients, however, have decreased expression of these central lipid regulators, and reduced abundance of several classes of phospholipids and cholesterol. In contrast, they show an increase in TAG and depend more on de novo cholesterol synthesis than HD T cells. We show that these metabolic alterations converge in dysfunctional lipid raft formation and reduced proliferation. Together, these data indicate an important and causal role of lipid alterations in CLL-induced T-cell dysfunction.

## Materials and methods

### Healthy donor and patient material

Peripheral blood mononuclear cells (PBMCs) were isolated and cryopreserved from the whole blood of CLL patients or buffy coats of age-matched HD (Supplementary Table 1) as described before [11]. Patients with FAO disorders were recruited during their visits to the hospital after receiving an information letter. FAOD were confirmed by genotype for all and enzyme activity for most patients (Supplementary Table 1). Written informed consent was obtained from all subjects in accordance with the Declaration of Helsinki and the study was approved by the medical ethics committee at Academic Medical Center, Amsterdam, the Netherlands (ethics approval number 2013/159).

### Cell culture

Cryopreserved PBMC were thawed and adjusted to a concentration of 3 × 10^6^ cells/ml, cultured in RPMI 1640 medium (11875093, Gibco) supplemented with 10% fetal calf serum, penicillin-streptomycin (15140-122, Thermo Fisher Scientific) and 2 mM glutamine (25030024, Gibco) at 37 °C and 5% CO_2_. An overview of all reagents used in the study can be found in Supplementary Table 2. T cells were stimulated using soluble αCD3 (clone 1XE, Sanquin, 1:10000) and αCD28 (clone 15E8, Sanquin, 1:1000) antibodies. In addition, PBMC were cultured in lipoprotein-deficient serum (LPDS) with or without re-added low-density lipoprotein (LDL) (composed of ApoA1 (0.06 mg/dl), ApoB (95.55 mg/dl), Tot. Chol (4.93 mmol/l), Triglycerides (0.47 mmol/l), HDL (0.73 mmol/l), LDLc (3.98 mmol/l)), or cultured in the presence of different inhibitors as specified in Supplementary Table 2. For membrane cholesterol depletion experiments, PBMCs were incubated with 2 mM methyl-β-cyclodextrin (MBCD) (C4555, Sigma) for 60 min prior to T-cell stimulation in the continued presence of MBCD.

### Flow cytometry

PBMCs were washed with ice-cold phosphate-buffered saline containing 0.5% bovine serum albumin (PBA) and EDTA. Afterward, cells were incubated with the indicated antibodies for surface staining for 20 min on ice. Specific information on the antibodies used in the study can be found in Supplementary Table 2. For intracellular staining, cells were washed with PBA, followed by fixation/permeabilization using the Fixation/Permeabilization Kit (554714, BD Biosciences) according to the manufacturer’s instructions. Mitochondrial FAO was measured by the fluorescence intensity of FAO Blue according to the manufacturer’s instructions (FDV-0033, Funakoshi). In short, cells were washed with PBS and incubated with 15 µM FAO Blue in serum-free RPMI media at 37 °C and 5% CO_2_. After incubation, cells were stained for surface proteins. Neutral lipid accumulation was measured by incubating samples with 1 μg/mL Bodipy^TM^493/503 (D3922, Thermo Fisher) in HBSS (14180046, Gibco) for 15 min at 37 °C and 5% CO_2_, followed by surface staining. Proliferation was assessed by labeling PBMC with 0.625 µM CellTrace Violet (C34557, Thermo Fisher Scientific) prior to in vitro T-cell stimulation.

Cells were acquired on an LSR Fortessa cytometer or a BD FACS Canto (BD Biosciences). For FACS sorting of CLL T cells, CD19 depletion was performed using magnetic-activated cell sorting (MACS) (130-042-401, 130-042-901, and 130-097-055, Miltenyi Biotec) according to manufacturer’s instructions, after which T cells were sorted using a FACSAriaIII (BD Biosciences). Data analysis was done using Flowjo v10 and v11 (TreeStar). Normalization of mean fluorescence intensity (MFI) from experiments was done in reference to either HD or unstimulated samples of the same donor.

### Confocal microscopy

After a 2-day culture of PBMCs with αCD3/αCD28 and MBCD when applicable, CD19 depletion was performed using MACS (130-042-401, 130-042-901 and 130-097-055, Miltenyi Biotec). For measurement of lipid aggregates, samples were stained with 5 μg/mL Bodipy^TM^ 493/503 (D3922, Thermo Fisher Scientific) in HBSS (14180046, Gibco) for 15 min at 37 °C and 5% CO_2_ prior to seeding. Isolated T cells from HD and CLL PBMC were then seeded on top of poly-lysine-L pre-coated coverslips on 24-well plates. Plates were allowed to rest at room temperature for 30 min to enable cells to attach, after which non-adherent cells were aspirated. Cells were fixed in 4% paraformaldehyde (047347.9 M, Life Technologies) for 10 min at room temperature. For lipid raft measurement, samples were stained with AF488-conjugated choleratoxin-B (CT-B) conjugate (C34775, Thermo Fisher Scientific) for 20 min on ice. Cells were then permeabilized and blocked using 0.5% Triton X-100 (9036-19-5, Sigma-Merck) and 1% BSA PBS, respectively. Primary antibodies against CD4 and CD8, both conjugated to AF594, and PLIN2, unconjugated (specified in Supplementary Table 2), were diluted in PBS and added at room temperature for 30 min. Cells were washed and stained with secondary antibody goat anti-mouse, AF647 in PBS for 30 min at room temperature for PLIN2 detection, if applicable. Cells were washed, and stained with DAPI (28718-90-3, Sigma-Merck) for 10 min. Then 5–10 µL of anti-fade fluorescence mounting medium (F6182, Sigma-Merck) was added to microscope slides, and coverslips were mounted on top, upside-down. Slides was allowed to dry for 15 min in the dark at room temperature and stored overnight at 4 °C. Imaging was performed no later than 24 h after mounting on a TCS SP8 X DLS confocal microscope (Leica Microsystems). Analysis of confocal images was done using QuPath v0.5.0 and ImageJ v1.50i. Co-localization of Bodipy^TM^ 493/503 and PLIN2 was assessed by quantification of fluorescence intensity across cells in ImageJ as gray values in spatial plots to assess co-occurrence in cross-sectional regions of interest (ROI). A maximum of one ROI was selected per T cell. In each T cell, the 50 pixels with the highest fluorescence intensity of Bodipy^TM^ 493/503 were selected, and the intensity of Bodipy^TM^ 493/503 and PLIN2 within the same pixel were plotted against each other. Correlation was calculated using linear regression analysis. Lipid raft mean and maximal fluorescence were calculated by quantifying CT-B fluorescence in T cells (selected based on CD4/CD8 staining) from multiple fields in QuPath v0.5.0.

### Lipidomics

CD19 depletion was performed on CLL and HD PBMC using MACS (130-050-301, Miltenyi Biotec) according to the manufacturer’s instructions and sorted, either directly after thawing or after a 2-day activation with αCD3/αCD28 in the original PBMC pool. In both conditions sorting was done using fluorescence-activated cell sorting (FACS) on a FACSAria^TM^ (BD). Two million CD4+ and CD8+ cells were pelleted per sample in 2 mL tubes and submitted to the Core Facility Metabolomics of Amsterdam UMC. Analysis of the lipidome was performed by liquid chromatography coupled to high-resolution mass spectrometry as previously described [33], with minor adjustments. Briefly: In each 2 mL tube with 2 million cells pellet, the following amounts of internal standards dissolved in 1:1 (v/v) methanol:chloroform were added: Bis(monoacylglycero)phosphate BMP(14:0)2 (0.2 nmol), Ceramide-1-phosphate C1P (d18:1/12:0) (0.127 nmol), D7-Cholesteryl Ester CE(16:0) (2 nmol), Ceramide Cer(d18:1/12:0) (0.118 nmol), Ceramide Cer(d18:1/25:0) (0.130 nmol), Cardiolipin CL(14:0)4 (0.1 nmol), Diacylglycerol DAG(14:0)2 (0.5 nmol), Glucose Ceramide GlcCer(d18:1/12:0) (0.126 nmol), Lactose Ceramide LacCer(d18:1/12:0) (0.129 nmol), Lysophosphatidicacid LPA(14:0) (0.1 nmol), Lysophosphatidylcholine LPC(14:0) (0.5 nmol), Lysophosphatidylethanolamine LPE(14:0) (0.1 nmol), Lysophosphatidylglycerol LPG(14:0) (0.02 nmol), Phosphatidic acid PA(14:0)2 (0.5 nmol), Phosphatidylcholine PC(14:0)2 (2 nmol), Phosphatidylethanolamine PE(14:0)2 (0.5 nmol), Phosphatidylglycerol PG(14:0)2 (0.1 nmol), Phosphatidylinositol PI(8:0)2 (0.5 nmol), Phosphatidylserine PS(14:0)2 (5 nmol), Sphinganine-1-phosphate S1P(d17:0) (0.124 nmol), Sphinganine-1-phosphate S1P(d17:1) (0.125 nmol), Ceramide phosphocholines SM(d18:1/12:0) (2.129 nmol), Sphingosine SPH(d17:0) (0.125 nmol), Triacylglycerol TAG(14:0)2 (0.5 nmol). One and a half mL 1:1 (v/v) methanol:chloroform was added before thorough mixing. Each sample was then centrifuged for 10 min at 14,000 rpm. Supernatant was transferred to a glass vial and evaporated under a stream of nitrogen at 60 °C. The residue was dissolved in 150 μL of 1:1 (v/v) methanol:chloroform. Lipids were analyzed using a Thermo Scientific Ultimate 3000 binary HPLC coupled to a Q Exactive Plus Orbitrap mass spectrometer. For normal phase separation, 2 μL of each sample were injected into a Phenomenex® LUNA silica, 250 × 2 mm, 5 µm 100 Å. Column temperature was held at 25 °C. Mobile phase consisted of (A) 85:15 (v/v) methanol:water containing 0.0125% formic acid and 3.35 mmol/L ammonia and (B) 97:3 (v/v) chloroform:methanol containing 0.0125% formic acid. Using a flow rate of 0.3 mL/min, the LC gradient consisted of: 10% A for 0–1 min, reach 20% A at 4 min, reach 85% A at 12 min, reach 100% A at 12.1 min, 100% A for 12.1–14 min, reach 10% A at 14.1 min, 10% A for 14.1–15 min. For reversed phase separation, 5 μL of each sample was injected into a Waters HSS T3 column (150 × 2.1 mm, 1.8 μm particle size). Column temperature was held at 60 °C. Mobile phase consisted of (A) 4:6 (v/v) methanol:water and (B) 1:9 (v/v) methanol:isopropanol, both containing 0.1% formic acid and 10 mmol/L ammonia. Using a flow rate of 0.4 mL/min, the LC gradient consisted of: 100% A at 0 min, reach 80% A at 1 min, reach 0% A at 16 min, 0% A for 16–20 min, reach 100% A at 20.1 min, 100% A for 20.1–21 min. MS data were acquired using negative and positive ionization using continuous scanning over the range of *m*/*z* 150 to *m*/*z* 2000. Data were analyzed using an in-house developed lipidomics pipeline written in the R programming language [34]. All reported lipids were normalized to corresponding internal standards according to lipid class. Lipid identification was based on a combination of accurate mass, (relative) retention times, fragmentation spectra, and the injection of relevant standards. Raw data will be deposited in MetaboLights (EMBL-EBI) and is available from the corresponding authors upon request.

### RNA sequencing

CD19 depletion was performed on CLL and HD PBMC using MACS (130-050-301, Miltenyi Biotec according to the manufacturer’s instructions and sorted, either directly after thawing or after a 2-day activation with αCD3/αCD28 in the original PBMC pool. In both conditions, sorting was done using FACS on a FACSAria^TM^ (BD). A minimum of one million cells were pelleted per sample, lysed and total RNA was isolated using the RNeasy mini kit (74106, Qiagen) according to the manufacturer’s protocol. RNA quality was assessed using a Fragment Analyzer and libraries were generated using the NEBNext Ultra II Directional RNA library prep kit for Illumina. Samples were barcoded and sequenced using a NovaSeq6000 (PE 150 bp). Quality of the sequencing data was assessed using FastQC. Raw FastQ files were aligned to the GRCh38 human genome using STAR v2.7.9a and the transcriptome.out.bam files were used as input for RSEM’s calculate expression (v1.3.3) to generate expected counts [35]. Normalized counts were generated with DEseq2 and plotted with pheatmap function, significant differential expression was determined at FDR < 0.05 [36]. The curated lipid-related gene lists used for transcription factor and pathway analysis can be found in Supplementary Table 3. Full list of significantly differentially expressed lipid-related genes can be found in Supplementary Table 4 (baseline) and 5 (day 2). Raw data will be deposited in the European Genome-phenome Archive (EGA) and is available from the corresponding authors upon request.

### Statistical analysis and data presentation

All statistical tests apart from lipidomics and RNAseq analyses were performed using Graphpad PRISM v10.1.2 and data are presented as mean ± standard error of the mean (SEM). For two-group comparisons, a paired/unpaired parametric two-tailed Student’s *t*-test was carried out, with the exception of the normalized counts of the RNAseq data, which were analyzed with the Mann–Whitney non-parametric *t*-test. For multiple group comparisons, two-way analysis of variance (ANOVA) tests were performed followed by Tukey multiple-comparisons test or Šídák multiple-comparisons tests with matched data. Differences were considered statistically significant if *p* < 0.05 and indicated as follows; *p < 0.05, **p < 0.01, ***p < 0.001, or ****p < 0.0001. The specific tests applied in each panel are indicated in figure legends. Illustrations were designed on https://biorender.com/. The results corresponding to CD4+ are presented in the main figures and CD8+ data are presented in supplementary figures unless stated otherwise.

## Results

### Extracellular cholesterol import is required for T-cell proliferation and is decreased in T cells from CLL patients

**Fig. 1:**
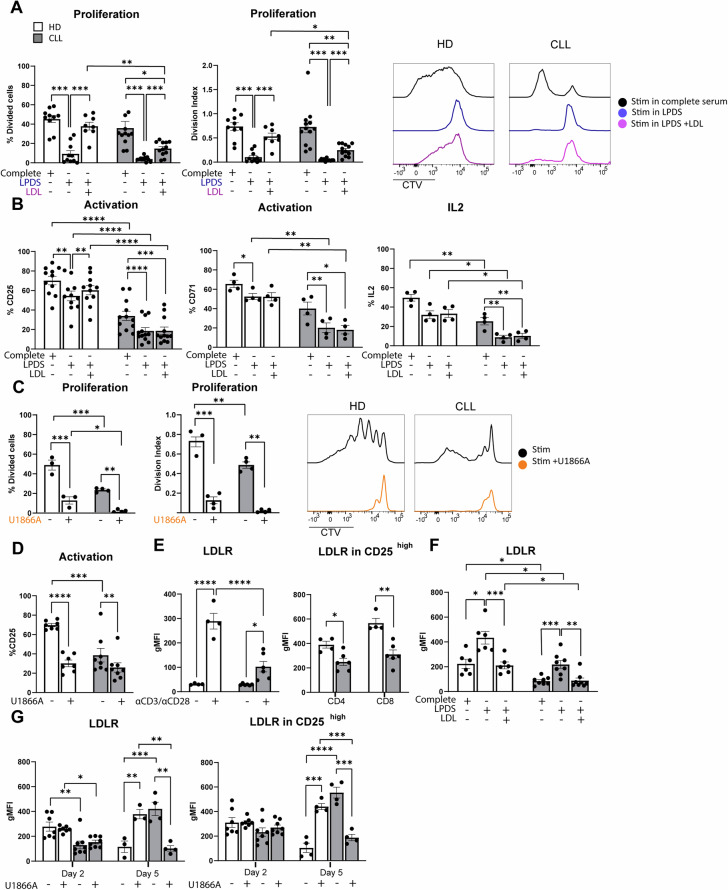
Extracellular cholesterol import is required for T-cell proliferation and is decreased in T cells from CLL patients. **A** PBMCs from healthy donors (HD) and chronic lymphocytic leukemia (CLL) patients were labeled with cell-trace violet (CTV) and stimulated with αCD3/αCD28 antibodies for 5 days under complete serum conditions, lipid deprivation (lipoprotein-deficient serum, LPDS) and LPDS supplemented with LDL. Proliferation of CD4+ T cells was measured as percentage divided cells (left) and division index (average number of cell divisions that a cell in the original population has undergone; right). Representative CTV histograms are shown (complete serum: black, LPDS: blue, and LPDS+ LDL: purple). **B** Expression of CD25, CD71 and IL-2 (intracellularly, after 4 h of Brefeldin treatment) was measured on CD4+ HD and CLL T cells after 2 days of stimulation in the same experimental conditions as in (**A**). **C** PBMCs from HD and CLL patients were stimulated with αCD3/αCD28 antibodies for 5 days in the presence or absence of the NPC1 inhibitor U18666A (10 µM). Proliferation of CD4+ T cells is shown as percentage divided cells (left) and division index (right). Representative CTV histograms are shown (αCD3/αCD28 only: black, αCD3/αCD28 + U1866A: orange). **D** Expression of CD25 was measured after 2 days of stimulation in the same experimental conditions as in (**C**). **E** Expression of LDLR was measured on CD4+ HD and CLL T cells after a 2-day stimulation with αCD3/αCD28 antibodies in total CD4+ T cells (left) and within CD4+ CD25^high^ T cells (right). **F** expression of LDLR was measured on CD4+ HD and CLL T cells in the same experimental conditions as in (**B**). **G** LDLR expression was measured on day 2 and day 5 upon PBMC stimulation in the presence or absence of the NPC1 inhibitor U18666A (10 µM) on CD4+ T cells (left) and within CD4+ CD25^high^ T cells (right). Data are presented as mean ± SEM and differences were analyzed with two-way repeated measures ANOVA with Tukey’s/Šidák’s multiple comparison test (**A**–**E** (left), **F**, **G**) or *t*-test (**E** (right)). **** = *p* < 0.0001; *** = *p* < 0.001; ** = *p* < 0.01; * = *p* < 0.05

T-cell proliferation necessitates cholesterol for the generation of new membranes, which can be acquired via biosynthesis and/or uptake. We hypothesized that these processes might be altered in T cells from CLL patients compared to HD, potentially contributing to the observed proliferation defects in CLL T cells. To assess this notion, PBMCs from CLL patients or age-matched HD were labeled with cell-trace violet (CTV) to be able to follow cellular divisions by flow cytometry, and stimulated with αCD3/28 soluble antibodies. Proliferation was assessed under two distinct culture conditions; In complete medium (i.e., medium containing lipoproteins) and in lipoprotein-deficient medium (LPDS). After 5 days, proliferation of T cells from both groups was compared. In line with previous reports [8, 13], proliferation of CLL T cells was reduced in complete medium conditions compared to HD, both in CD4+ and CD8+ T cells (Supplementary Fig. 1A). T-cell proliferation was fully abrogated in the LPDS condition in HD and CLL CD4+ (Fig. 1A) and CD8+ T cells (Supplementary Fig. 1B), indicating a clear dependency on exogenous cholesterol for proliferation, which was evident in the percentage of dividing cells and in the average number of cell divisions that a cell in the original population has undergone (division index). A primary route for cells to acquire exogenous cholesterol relies on LDL uptake by the LDL receptor (LDLR) [37]. We therefore tested whether supplementation of LDL restored proliferation in LPDS-cultured cells. Our results demonstrate that the impaired proliferation of HD and CLL cells in LPDS could be rescued by LDL supplementation, albeit the effect being smaller in CLL cells (Fig. 1A, Supplementary Fig. 1B).

Differences in T cell activation between HD and CLL T cells have been previously reported [8] and can contribute to different proliferation rates. Thus, we evaluated by flow cytometry the expression of several markers of T-cell activation and effector function in the lipid deprivation setting. The evaluated markers were IL-2 receptor (CD25), transferrin receptor 1 (CD71), cyclic ADP ribose hydrolase (CD38), the early activation marker CD69, intracellular interleukin 2 (IL-2), and granzyme B (GZMB). CD25 levels were found reduced in stimulated T cells from CLL patients, as expected [11]. A reduction in CD25 expression was observed in the LPDS condition in both HD and CLL CD4+ and CD8+ T-cells (Fig. 1B, Supplementary Fig. 1C). CD71 was also decreased in the LPDS condition in CD4+ T cells from both groups (Fig. 1B), and in CD8+ T cells from CLL patients (Supplementary Fig. 1C). Intracellular IL-2 was decreased in the LPDS condition in HD only in the CD8+ compartment, and in CLL in both CD4+ and CD8+ (Fig. 1B, Supplementary Fig. 1C). Furthermore, surface levels of CD38 and CD69 were decreased in LPDS conditions only in T cells from CLL patients, but not in HD (Supplementary Fig. 1D). Taken together, the effects of extracellular cholesterol depletion on T-cell activation and effector markers were much more pronounced in T cells from CLL patients than in HD, possibly indicating a lack of compensatory mechanisms to maintain cholesterol levels in these cells. Except for a small increase in CD25 expression in HD, LDL supplementation did not restore the expression of activation or effector markers, suggesting that lipoprotein availability is not directly linked to activation status in T cells.

Following its uptake, LDL is trafficked to the lysosome where cholesterol egress to other cellular compartments is initially coordinated by the cholesterol transporter Niemann-Pick C1 protein (NPC1). As LDL uptake is a primary way for cells to acquire exogenous cholesterol, we reasoned that attenuated cholesterol availability was the limiting factor for T-cell proliferation in the LPDS setting. To further substantiate this claim, we inhibited NPC1 with the small molecule U18661A [38]. Treating cells with U18661A mimics sterol depletion without altering the medium composition [39]. Similar to the results obtained with LPDS medium, the percentage of proliferating cells and division index of HD and CLL T cells was reduced when NPC1 was inhibited (Fig. 1C, Supplementary Fig. 1E). In line with this, T-cell activation was reduced when cultured in the presence of U18661A (Fig. 1D, Supplementary Fig. 1F).

To further investigate whether defects in LDL uptake could be, at least in part, the basis of decreased proliferation in CLL T cells, the surface abundance of LDLR was assessed by flow cytometry. This analysis showed that HD T cells robustly increased the abundance of surface LDLR following T-cell stimulation, while this response was severely impaired in CLL T cells (Fig. 1E left, Supplementary Fig. 1G). In order to rule out the differences in activation status between HD and CLL patients as a confounding factor, LDLR levels were also analyzed within the highly activated T-cell population (CD25^high^) in both groups (gating strategy example in Supplementary Fig. 1H). Also in the CD25^high^ sub-population, LDLR levels were reduced in CLL compared to HD T cells (Fig. 1E right), indicating that reduced surface LDLR is an inherent lesion of CLL T cells that is not dependent on the degree of activation. Expression of *LDLR* is primarily regulated by sterol levels through the SREBP transcriptional pathway [37, 40]. Thus, we next assessed LDLR levels in response to a 2-day stimulation in LPDS medium. Lipid deprivation-induced LDLR expression in HD T cells (Fig. 1F, Supplementary Fig. 1I) was similar to responses previously described in hepatocytes and macrophages [41–43]. As expected, LDL supplementation decreased the level of LDLR to that measured in the control condition (Fig. 1F, Supplementary Fig. 1I). The same pattern was observed in CLL T cells, revealing that despite decreased LDLR levels, sterol sensing mechanisms are functional in these cells (Fig. 1F, Supplementary Fig. 1I). However, the dynamics of LDLR regulation upon stimulation in the presence of U18661A were divergent. In HD, LDLR was only increased after 5 days of stimulation in the presence of U18661A (Fig. 1G left, Supplementary Fig. 1J left), when T-cell activation levels reached their peak (Supplementary Fig. 1K [44]). In contrast, LDLR expression was decreased in CLL T cells at day 5 in the presence of U18661A, compared to their control stimulated counterparts (Fig. 1G left, Supplementary Fig. 1J left). Differences in LDLR levels upon U1886A were also observed within the CD25 ^high^ sub-population at day 5 (Fig. 1G right, Supplementary Fig. 1J right), indicating that CLL T cells likely lack additional determinants to properly sense cholesterol deficiency upon U18661A exposure. Altogether, these results show that T cells are dependent on exogenous cholesterol for expansion upon TCR-mediated activation. Furthermore, CLL T cells not only have reduced expression of LDLR but also fail to fully adapt to cholesterol deprivation.

### Key transcriptional programs governing lipid metabolism are downregulated in CLL T cells

**Fig. 2:**
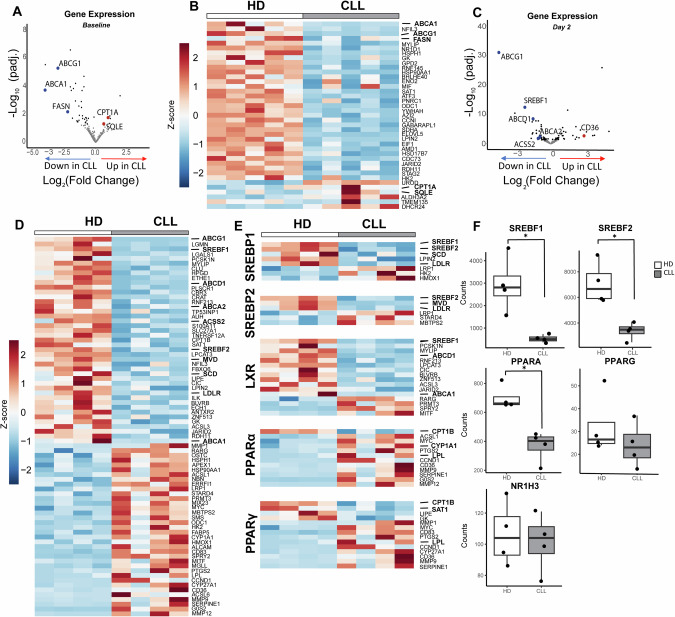
Key transcriptional programs governing lipid metabolism are downregulated in CLL T cells. CD4+ and CD8+ T cells of HD and CLL patients were FACS-sorted and subjected to RNA sequencing, either (**A**, **B**) at baseline or (**C**–**F**) after a 2-day stimulation with αCD3/αCD28 antibodies. To specifically analyze the main transcription factor families regulating lipid metabolism and their targets, a list of 452 genes was compiled using publicly available gene sets (Supplemental Table 3). Expression of these genes is visualized in volcano plots and heatmaps. **A, C** In the volcano plots, differentially expressed genes between CLL and HD T cells (*p*adj. ≤ 0.05) are depicted in black dots and, among them, specific genes of interest are highlighted in blue (downregulated in CLL) or red (upregulated in CLL). **B**, **D** Heatmaps showing *Z*-scores of the significantly differentially expressed genes between the two groups (*p*adj. < 0.05). Genes of interest are highlighted in bold. **E** Heatmap of the expression of differentially expressed SREBP1, SREBP2, PPARα, PPARγ, and LXR target genes in CLL compared to HD T cells (*p*adj. ≤ 0.05) after a 2-day stimulation with αCD3/αCD28 antibodies. Genes of interest are highlighted in bold. **F **Boxplots showing DESeq2 normalized counts of *SREBF1, SREBF2, PPARA, PPARG*, and *NIH13* in CLL and HD T cells after a 2-day stimulation. Differences in (**F**) were analyzed with Mann–Whitney test. * = *p* < 0.05

Lipid metabolism in T cells is subject to tight (post-) transcriptional regulation [24]. Herein, the SREBPs, PPARs, and the ligand X receptors (LXR) play a crucial role [24]. To uncover transcriptional differences that may be at the basis of the proliferation defects in CLL T cells, the transcriptome of HD and CLL T cells was compared at baseline and after 2 days of TCR stimulation. To avoid differences in subset skewing between HD and CLL as confounders in these analyses, samples were matched for CD4, CD8, and subset distribution (Supplementary Fig. 2A, B). In addition, selected samples had comparable expression levels of CD25 upon activation, and an equal CD4 to CD8 distribution after sorting (Supplementary Fig. 2C). In order to specifically analyze the T-cell transcriptome, PBMCs (either at baseline or after 2 days of stimulation with αCD3/28 soluble antibodies) were subjected to CD19 depletion by MACS followed by sorting of viable CD4+ and CD8+ T cells. Samples were pelleted, and mRNA was extracted and subjected to RNA sequencing. Both at baseline and upon in vitro stimulation, CLL and HD T cells clustered separately in a principal component analysis, indicating they have distinct transcriptomic signatures (Supplementary Fig. 2D). To specifically analyze whether the expression of lipid genes was different between the two groups, we compiled a list of 452 lipid-related genes using publicly available gene sets of target genes regulated by SREBPs, PPARs, and LXR, and specific lipid pathways (FA metabolism and cholesterol metabolism) (Supplementary Table 3). Out of the 452 genes, 119 were differentially expressed between HD and CLL T cells at baseline, of which the majority were reduced in CLL T cells (Fig. 2A, Supplementary Table 4). Significantly downregulated genes included the LXR-regulated cholesterol efflux transporters *ABCA1* and *ABCG1*, as well as FA synthase (*FASN*). Upregulated genes included carnitine palmitoyl transferase I (*CPT1A*) and squalene epoxidase (*SQLE*), a rate-limiting enzyme in the cholesterol biosynthetic pathway (Fig. 2B), indicating that T cells from both groups already differed in the regulation of some specific lipid genes prior to stimulation. However, we did not identify homogenous up/downregulation of specific transcription factor signatures at baseline.

Upon stimulation, 74 out of the 452 genes were differentially expressed. Among the most significantly downregulated genes in CLL T cells, we found FA and cholesterol transporters (*ABCG1*, *ABCD1*, and *ABCA2*) and *SREBF1/2* (SREBP1/2) (Fig. 2C, D, Supplementary Table 5). Accordingly, multiple target genes of SREBP1/2 were downregulated in CLL T cells upon stimulation, including Stearoyl-CoA Desaturase (*SCD*), an SREBP1 target involved in FA desaturation, *LDLR*, an SREBP2 target we previously found decreased at the protein level (Fig. 1E, Supplementary Fig. 1G), and mevalonate diphosphate decarboxylase (*MVD*), an SREBP2 target involved in cholesterol synthesis (Fig. 2D, E). Contrarily, most PPARα target genes were upregulated in CLL with the exception of *CPT1B*, involved in FA transport in the mitochondria (Fig. 2E), while expression of *PPARA* itself was significantly lower in CLL T cells (Fig. 2F). Expression of the transcription factors *PPRG* (PPARγ) and *NR1H3* (LXR) was not significantly different between both groups (Fig. 2F), however, most LXR target genes were downregulated in CLL T cells, and PPARγ target genes showed a mixed response (Fig. 2E). Analysis of gene expression by functional pathways revealed that CLL T cells exhibited an overall decrease in the expression of genes involved in cholesterol biosynthesis and FA metabolism upon stimulation (Supplementary Fig. 2E). Collectively, these findings demonstrate that the key transcriptional programs governing lipid metabolism are dampened in CLL T cells compared to healthy T cells.

**Fig. 3:**
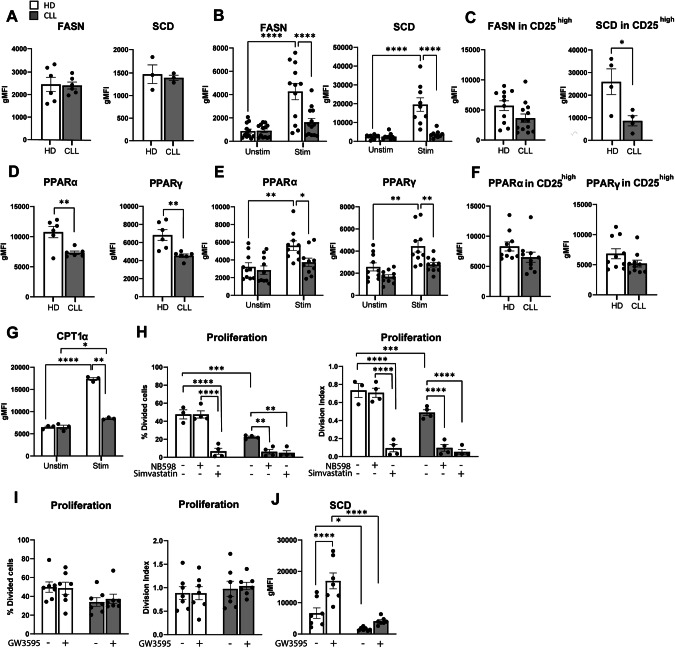
Essential lipid metabolism proteins are downregulated in CLL T cells, which portrays increased dependency on de novo cholesterol biogenesis. **A** Levels of FASN and SCD were measured on CD4+ T cells from HD and CLL patients at baseline, **B** after a 2-day stimulation with αCD3/αCD28, and **C** within CD4+ CD25^high^ T cells by flow cytometry. **D** Expression of PPARα and PPARγ was measured on CD4+ T cells from HD and CLL patients at baseline, **E** after a 2-day stimulation with αCD3/αCD28, and **F** within CD4+ CD25^high^ T cells. **G** Expression of CPT1α was measured on CD4+ T cells from HD and CLL patients after a 2-day stimulation with αCD3/αCD28. **H** PBMCs from HD and CLL patients were labeled with CTV and stimulated with αCD3/αCD28 antibodies for 5 days in the presence or absence of the squalene inhibitor NB598 (10 µM) or Simvastatin (10 µM). Proliferation of CD4+ T cells is shown as percentage divided cells (left) and division index (right). **I** PBMCs from HD and CLL patients were labeled with CTV and stimulated with αCD3/αCD28 antibodies for 5 days in the presence or absence of the LXR agonist GW3695 (1 µM). Proliferation of CD4+ T cells is shown as percentage divided cells (left) and division index (right). **J** Expression of SCD at day 2 was measured under the same experimental conditions as in (**I**). Data are presented as mean ± SEM and differences were analyzed with *t*-tests (**A**, **C**, **D**, **F**) or two-way repeated measures ANOVA with Tukey’s/Šidák’s multiple comparison test (**B**, **E**, **G**, **H**, **I**, **J**). **** = *p* < 0.0001; *** = *p* < 0.001; ** = *p* < 0.01; * = *p* < 0.05

We next examined the relevance of our observation of lower levels of key transcription regulators upon stimulation in CLL T cells by measuring protein levels of FASN and SCD by flow cytometry. These enzymes are essential for FA synthesis and desaturation, respectively, and are both well-established downstream targets of SREBP1. FASN and SCD were equally abundant in CLL and HD T cells at baseline (Fig. 3A, Supplementary Fig. 3A), but CLL T cells failed to upregulate them upon TCR activation (Fig. 3B, Supplementary Fig. 3B). Analysis of the sub-population of CD25^high^ T cells revealed a trend towards decreased levels of FASN and a significant reduction in SCD expression in CLL T cells compared to HD T cells (Fig. 3C, Supplementary Fig. 3C). This indicates that defects in SREBP1 signaling are inherent in T cells from CLL patients and not a product of decreased activation levels.

Decreased protein levels of PPARγ and PPARα were observed in CLL T cells at baseline (Fig. 3D, Supplementary Fig. 3D). Upon activation, expression of these proteins increased significantly in HD T cells but not in CLL T cells (Fig. 3E, Supplementary Fig. 3E), including CD25^high^ cells (Fig. 3F, Supplementary Fig. 3F), where a statistically significant reduction of protein levels was observed in the CD8+ compartment compared to healthy CD8+ cells (Supplementary Fig. 3F). Accordingly, significantly lower levels of the PPARα target CPT1α were detected in CLL T cells upon stimulation, compared to HD T cells (Fig. 3G, Supplementary Fig. 3G).

In conclusion, CLL T cells exhibited impaired upregulation of key transcription factors governing lipid metabolism, especially SREBP1 and 2, PPARα and PPARγ and its target genes, upon TCR stimulation, implying reduced capacity for FA and cholesterol synthesis and limited use of lipids for energy production.

### T cells from CLL patients have increased dependency on de novo cholesterol biogenesis compared to healthy T cells

Cholesterol homeostasis involves uptake (through LDLR), efflux, and de novo biogenesis, which is even more relevant when uptake is impaired. The SCAP-SREBP2 complex in the endoplasmic reticulum (ER) senses low sterol levels, triggering SREBP2 proteolysis [45]. Cleaved active SREBP2 translocates to the nucleus and increases the transcription of genes of the mevalonate pathway, which facilitates the synthesis of cholesterol and its derivatives [45, 46]. Transcriptomic analyses showed decreased expression of LDLR and MVD (Fig. 2E), which catalyzes one of the essential steps in cholesterol biogenesis in the mevalonate pathway. To compare the activity of this pathway between HD and CLL T cells, we targeted two distinct enzymatic steps and evaluated proliferation, using a SQLE inhibitor (NB598) and an HMG-CoA reductase inhibitor (simvastatin) during PBMC simulation. It is important to note that simvastatin has previously been reported to not only halt cholesterol biosynthesis but to also inhibit the isoprenoid branch of the mevalonate pathway, disturbing membrane composition and inhibiting protein prenylation in T cells [47]. NB598 dampened proliferation only in CLL T cells, whilst simvastatin significantly reduced it in both HD and CLL T cells (Fig. 3H, Supplementary Fig. 3H). These results suggest a higher dependency on de novo cholesterol biogenesis in CLL T cells compared to HD. We speculate that healthy T cells compensate for the inhibition of SQLE by increasing exogenous cholesterol uptake through LDLR, whilst we have shown that CLL T cells are not able to equally do so upon cholesterol deprivation (Fig. 1F, G, Supplementary Fig. 1I, J).

While SREBP2 controls cholesterol uptake and synthesis, LXR regulates cholesterol efflux. To investigate its relevance in cholesterol homeostasis in T cells, we used the LXR agonist GW3695. Increase of cholesterol efflux by GW3695 did not affect T-cell proliferation in neither HD or CLL cells (Fig. 3I, Supplementary Fig. 3I), which is different from what was previously reported in mice [48]. Nevertheless, levels of SCD, which is indirectly regulated by LXR through SREBP1, were strongly upregulated by GW3695 in HD T cells but not in CLL (Fig. 3J, Supplementary Fig. 3J). This indicates that the axis LXR-SREBP1 is more active in HD T cells than in CLL. In summary, these data demonstrate a tight link between cholesterol availability and proliferation in T-cells, which is compromised at the levels of uptake and synthesis in CLL.

### The organization of lipids in the cytoplasm is different in T cells from CLL patients compared to HD

**Fig. 4:**
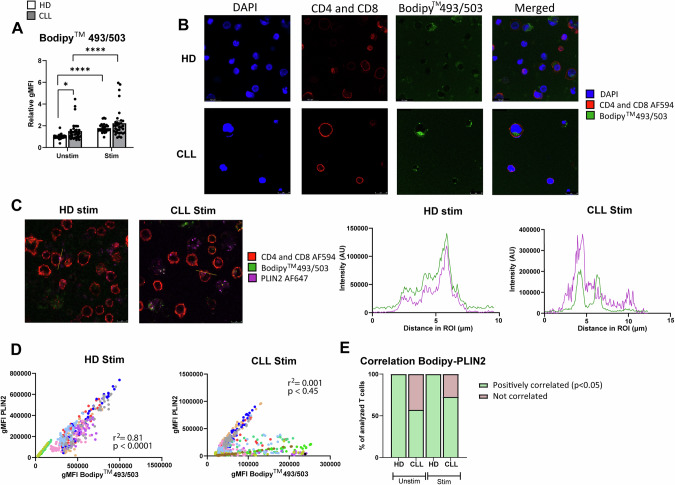
The organization of lipids in the cytoplasm is different in T cells from CLL patients compared to HD. **A** PBMCs from HD and CLL patients were stimulated with αCD3/αCD28 antibodies for 2 days. Neutral lipid accumulation was quantified using Bodipy^TM^493/503 staining by flow cytometry on CD4+ T cells. Raw gMFI values were normalized to unstimulated HD samples in each independent experiment. **B** Upon culturing in the same experimental conditions as in (**A**), samples were subjected to CD19 MACS depletion and immunofluorescence was performed, first staining with Bodipy^TM^493/503 and then with antibodies against CD4+ and CD8+ (both conjugated to AF549). DAPI was used as nuclear staining. Samples were imaged with confocal microscopy. Representative images of a stimulated HD and CLL sample are shown. **C** Upon culturing in the same experimental conditions as in (**A**), immunofluorescence was performed, first staining with Bodipy^TM^493/503 and then with antibodies against CD4+ and CD8+ (both conjugated to AF549) and PLIN2 (unconjugated). A secondary antibody goat anti-mouse conjugated to AF647 was used to detect PLIN2, and DAPI was used as nuclear staining. Regions of interest (ROI) were manually selected based on Bodipy^TM^493/503. Fluorescence intensity of Bodipy^TM^493/503 and PLIN2 was quantified in ImageJ as gray values throughout each ROI and plotted in spatial plots to assess co-occurrence in a cross-sectional ROI. Representative images with ROIs highlighted in yellow are provided (left). Representative spatial plots of fluorescence intensity from a stimulated HD and CLL samples are shown (right). **D** In each ROI, the 50 pixels with the highest Bodipy^TM^493/503 fluorescence were selected. Intensity of Bodipy^TM^493/503 and PLIN2 within the same pixel were plotted against each other and correlation was calculated. Data from all ROIs analyzed in stimulated cells are shown. Every dot represents one pixel and every color corresponds to one ROI. **E** The percentage of T cells with significant positive correlation between Bodipy^TM^493/503 and PLIN2 was calculated in each condition (*p* < 0.05). Data are presented as mean ± SEM (**A**) and differences were analyzed with two-way repeated measures ANOVA with Tukey’s/Šidák’s multiple comparison test (**A**) or linear regression analysis (**D**). **** = *p* < 0.0001; * = *p* < 0.05

Decreased SREBP1/2 signaling upon TCR stimulation in CLL implies reduced capacity for FA and cholesterol synthesis and thus we hypothesized that CLL T cells would exhibit reduced intracellular lipid content. In order to analyze this, the fluorescent dye Bodipy^TM^493/503 was used. PBMCs were stimulated for 2 days and subsequently incubated in the presence of Bodipy^TM^493/503, which diffuses inside cells, binds to neutral lipids, and can be detected by flow cytometry. Contrary to our expectations, lipid storage was increased in CLL T cells both in unstimulated and stimulated conditions, compared to HD, which was especially notable within the CD8+ T-cell subset (Fig. 4A, Supplementary Fig. 4A). Monocytes, which are known to accumulate substantial amounts of lipids in their cytoplasm [23] were used as reference (Supplementary Fig. 4B). Confocal microscopy further validated the presence of lipid aggregates based on Bodipy^TM^493/503 staining in the cytoplasm of T cells (Fig. 4B), supporting our findings by flow cytometry.

Lipid droplets, primarily characterized in adipocytes, typically express perilipin-2 (PLIN2) on their surface [23]. To investigate whether the identified neutral lipid aggregates in T cells were organized in lipid droplets, we conducted confocal microscopy to assess co-localization of Bodipy^TM^493/503 with PLIN2. PBMCs were stimulated for 2 days and subjected to CD19 depletion by MACS before staining of Bodipy^TM^493/503, PLIN2 and DAPI for confocal analyses (Supplementary Fig. 4C, D). Cross-sectional analysis of T cells in both HD and CLL PBMCs revealed strong co-localization between Bodipy^TM^493/503 and PLIN2 in HD T cells (Fig. 4C, Supplementary Fig. 4E), particularly in stimulated samples, indicating that all lipid aggregates in these cells were lipid droplets (PLIN2+). In contrast, areas positive for Bodipy^TM^493/503 were not always PLIN2-positive in CLL T cells (Fig. 4C, Supplementary Fig. 4E), suggesting a different organization of neutral lipids in the cytoplasm. Linear regression analysis of Bodipy^TM^493/503 and PLIN2 signal within multiple cells showed significant correlation in HD T cells, in both stimulated (Fig. 4D) and unstimulated conditions (Supplementary Fig. 4F). This positive correlation was found in all T cells analyzed in HD (Fig. 4E, Supplementary Fig. 4G). In CLL, two different T-cell populations were found, one where co-localization of Bodipy^TM^493/503 and PLIN2 occurred (57.1% in the unstimulated, and 72.7% in the stimulated samples), and another where no co-localization was present. Even within cells where co-localization was present, PLIN2-negative lipid aggregates were observed (Fig. 4D, Supplementary Fig. 4F, G).

### The lipidome of CLL T cells is characterized by low cholesterol and phospholipids, and accumulation of triglycerides, compared to healthy T cells

To compare the lipid composition of HD and CLL T cells, we performed untargeted mass spectrometry-based lipidomic analysis at baseline and in samples stimulated for 2 days from the same HD and patients used in the RNA sequencing experiments. This approach enabled a direct comparison between lipid profiles and gene expression patterns, enhancing our understanding of metabolic alterations in CLL T cells.

**Fig. 5:**
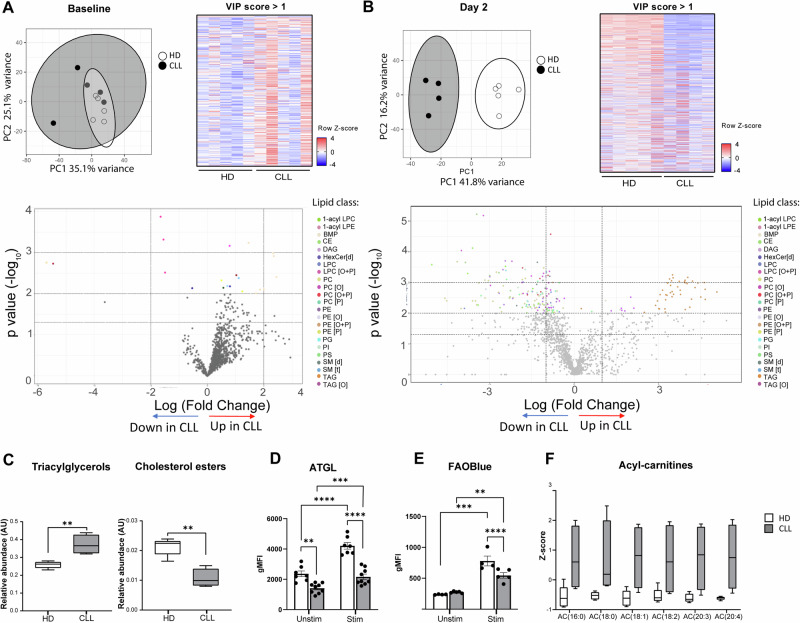
The lipidome of CLL T cells is characterized by low cholesterol and phospholipids, and accumulation of triglycerides, compared to healthy T cells. CD4+ and CD8+ T cells of HD and CLL patients were FACS-sorted and subjected to liquid chromatography-mass spectrometry (LC-MS)-based lipidomics (**A**) at baseline or (**B**) after 2-day stimulation with αCD3/αCD28 antibodies. Abundance of each lipid molecule was normalized by internal standards and protein amount (**A**) or total lipidome pool (**B**). Results are visualized as principal component analysis plots, volcano plots, and heatmaps of differentially abundant lipid species. In the volcano plots, the three horizontal dotted lines indicate *p* values of 0.05, 0.01, and 0.001, respectively. The two vertical dotted lines indicate log2 (fold change) of −2 and 2, respectively. Lipid molecules with significantly different abundance in CLL T cells compared to HD T cells (*p* ≤ 0.01) are colored according to the lipid class they belong to. For heatmap representation of lipidomics data, a cutoff for variable importance in projection (VIP) score >1 from all lipid molecules analyzed was defined. Abundance of the different lipid classes relative to the total lipids sum is shown in Supplementary Fig. 5A, B. **C** Relative abundance of triacylglycerols and cholesterol esters after a 2-day stimulation with αCD3/αCD28 antibodies is plotted separately. **D** PBMC from HD and CLL patients were stimulated for 2 days with αCD3/αCD28 antibodies and protein levels of ATGL were measured. **E** FAO was assessed by flow cytometry on CD4+ T cells by FAOBlue in the same experimental conditions as in (**D**). **F** Abundance of acylcarnitines after a 2-day stimulation with αCD3/αCD28 antibodies is plotted separately. Data are presented as mean ± SEM (**C**–**F**) and differences were analyzed with *t*-test (**C**) or two-way repeated measures ANOVA with Tukey’s/Šidák’s multiple comparison test (**D**–**F**). **** = *p* < 0.0001; *** = *p* < 0.001; ** = *p* < 0.01; * = *p* < 0.05. PC phosphatidylcholine, PC[O] alkylphosphatidylcholine, PC[P] alkenylphosphatidylcholine, PC[O + P] alkyl/alkenylphosphatidylcholine, LPC lysophosphatidylcholine, LPC[O] alkyllysophosphatidylcholine, LPC[P] alkenyllysophosphatidylcholine, LPC[O + P] alkyl/alkenyllysophosphatidylcholine, PE phosphatidylethanolamine, PE[P] alkenylphosphatidylethanolamine, PE[O] alkylphosphatidylethanolamine, PE[O + P] alkyl/alkenylphosphatidylethanolamine, LPE lysophosphatidylethanolamine, LPE[P] alkyllysophosphatidylethanolamine, LPE[O + P] alkyl/alkenyllysophosphatidylethanolamine, PI phosphatidylinostiol, BMP bis(monoacylglycero)phosphate, PG phosphatidyglycerol, PS phosphatidylserine, CL cardiolipin, SM[d] sphingomyeline, SM[t] hydroxysphingomyeline, SPH[d] sphingosine, GM3 monosialdihexosylganglioside, Cer[d] ceramide, HexCer[d] hexosylceramide, Hex2Cer[d] dihexosylceramide AC acylcarnitine, TAG triacylglycerol, TAG[O] alkyltriacylglycerol, DAG diacylglycerol, CE cholesterol ester, FA fatty acid

In order to specifically analyze the T-cell lipidome, PBMCs (either at baseline or after 2 days of stimulation with αCD3/28 soluble antibodies) were subjected to CD19 depletion by MACS, followed by sorting of viable CD4+ and CD8+ T cells. Samples were pelleted and subjected to lipidome extraction and liquid chromatography-mass spectrometry (LC-MS) analysis. Principal component analysis of lipidomic profiles at baseline revealed no global differences between HD and CLL T cells on the first 2 principal components (Fig. 5A). However, a general trend to increased lipid abundance was observed in CLL T cells across all lipid classes (Supplementary Fig. 5A). This trend was particularly evident among lipids with a variable importance in projection (VIP) higher than 1, i.e., the most different between the two groups (Fig. 5A). These findings align with the accumulation of neutral lipids observed in CLL T cells at baseline (Fig. 4A, Supplementary Fig. 4A).

After 2 days of stimulation, a clear separation in lipid composition was identified between CLL and HD T cells (Fig. 5B). The majority of lipids with a VIP score higher than 1 were lower in abundance in CLL T cells compared to HD (Fig. 5B). Grouping lipids by structural similarity revealed decreased relative abundance of most classes in CLL T cells, including essential phospholipids that are part of cellular membranes, such as phosphatidylcholine (PC) and sphingomyelin (SM) (Fig. 5B, Supplementary Fig. 5B). Notably, CE were also found decreased in CLL T cells (Fig. 5C, Supplementary Fig. 5B), corroborating our previous findings of a reduced SREBP2 signature in these cells. Conversely, TAG were significantly increased in CLL T cells (Fig. 5C, Supplementary Fig. 5B). This suggests that the neutral lipid accumulations previously detected by flow cytometry and confocal imaging (Fig. 4) likely have different compositions in CLL T cells (predominantly TAG) versus HD (predominantly CE). In summary, lipidomic analyses revealed a distinct lipid profile in stimulated CLL T cells characterized by cholesterol deficiency and TAG accumulation. This pattern may result from decreased TAG utilization in CLL T cells.

Lipolysis, the process of harnessing energy reserves from stored TAG, is crucial for cellular metabolism but has not been extensively studied in T cells [46]. This tightly regulated process is dependent on several enzymes, including the adipose triglyceride lipase (ATGL), which catalyzes the first reaction [23]. Our findings of increased neutral lipid accumulation in CLL T cells coupled with decreased expression of enzymes involved in de novo synthesis and uptake of FA and cholesterol presents a paradox. We hypothesized that lipid accumulation could be due to impaired lipolysis.

To investigate this hypothesis, we assessed ATGL levels in HD and CLL T cells by flow cytometry. Expression of ATGL was found decreased in CLL as compared to HD, in both unstimulated CD4+ and stimulated CD4+ and CD8+ T cells (Fig. 5D, Supplementary Fig. 5C). To evaluate the relevance of ATGL in regulating lipolysis in T cells, HD T cells were treated with the ATGL-inhibitor Acipimox, which resulted in increased T-cell lipid content in T cells in both groups (Supplementary Fig. 5D). This effect was more clear in HD, in accordance with higher ATGL levels in this group. Lipid content was also increased in monocytes (Supplementary Fig. 5D as expected based on previous literature [23]. These results suggest that low ATGL might limit the capacity for lipolysis of CLL T cells and lead to TAG accumulation.

Another plausible explanation for the observed accumulation of lipids in CLL T cells could be a reduced capacity for FAO. To investigate this, we incubated PBMCs after a 2-day incubation with FAO Blue, a modified FA containing a coumarin group that is metabolized as a medium-chain FA and emits fluorescence upon the release of the coumarin group after four FAO cycles, which can be detected by flow cytometry [49]. Lower mitochondrial FAO activity was found in CLL T cells upon stimulation as compared to HD (Fig. 5E, Supplementary Fig. 5E). This observation aligns with the reduced levels of CPT1α identified in stimulated CLL T cells (Fig. 3G, Supplementary Fig. 3G). Furthermore, lipidomic analysis revealed increased acylcarnitines (AC) in CLL T cells (Fig. 5F, Supplementary Fig. 5B). AC are substrates of FAO formed during the mitochondrial membrane transport of FA mediated by CPT1α [50]. Accumulation of AC provides additional support for defective or slower FAO in CLL T cells.

Our findings demonstrate that human T cells have the capacity to store neutral lipids in the form of lipid droplets. However, in CLL T cells a fraction of neutral lipids is stored in a PLIN2-negative compartment, indicating a distinct lipid storage mechanism compared to HD T cells. Furthermore, our results show that CLL T cells do not efficiently utilize their stored lipids as a fuel for mitochondrial FAO and energy production. It remains to be determined whether defects in neutral lipid storage/utilization and FAO contribute to the decreased activation and proliferation of CLL T cells upon TCR ligation.

### FAO is essential for T-cell activation, while membrane lipid composition determines lipid raft formation and proliferation

The specific branches of lipid metabolism required for distinct T-cell functions, such as activation and proliferation, remain to be fully elucidated. We have identified lipid defects in CLL, primarily characterized by decreased CE content and lower rates of FAO. Both of these alterations could potentially impact T-cell processes. To gain insight into which pathway contributes to specific T-cell functions, we assessed T-cell responses in patients with genetic FAO defects and compared them to T cells from CLL patients and HD.

We analyzed PBMCS from five patients with FAO disorders; one with a mutation in the *CPT1A* gene (OMIM 600528, causing CPT1α deficiency), two with mutations in *CPT2* gene (OMIM 600650), and two with mutations in *HADHA*, one of the two genes encoding for the mitochondrial trifunctional protein (MTP) (OMIM 600890, causing MTP deficiency). Detailed patient information is provided in Supplementary Table 1. Biochemically, FAO disorders are characterized by an accumulation of AC that cannot be metabolized through FAO. This well-established diagnostic tool is also the method by which neonatal screening for these disorders is performed [51, 52]. As we also found increased AC levels in CLL T cells, T-cell activation and proliferation upon TCR triggering were evaluated in these five patients to elucidate the specific roles of FAO in these processes [53].

**Fig. 6:**
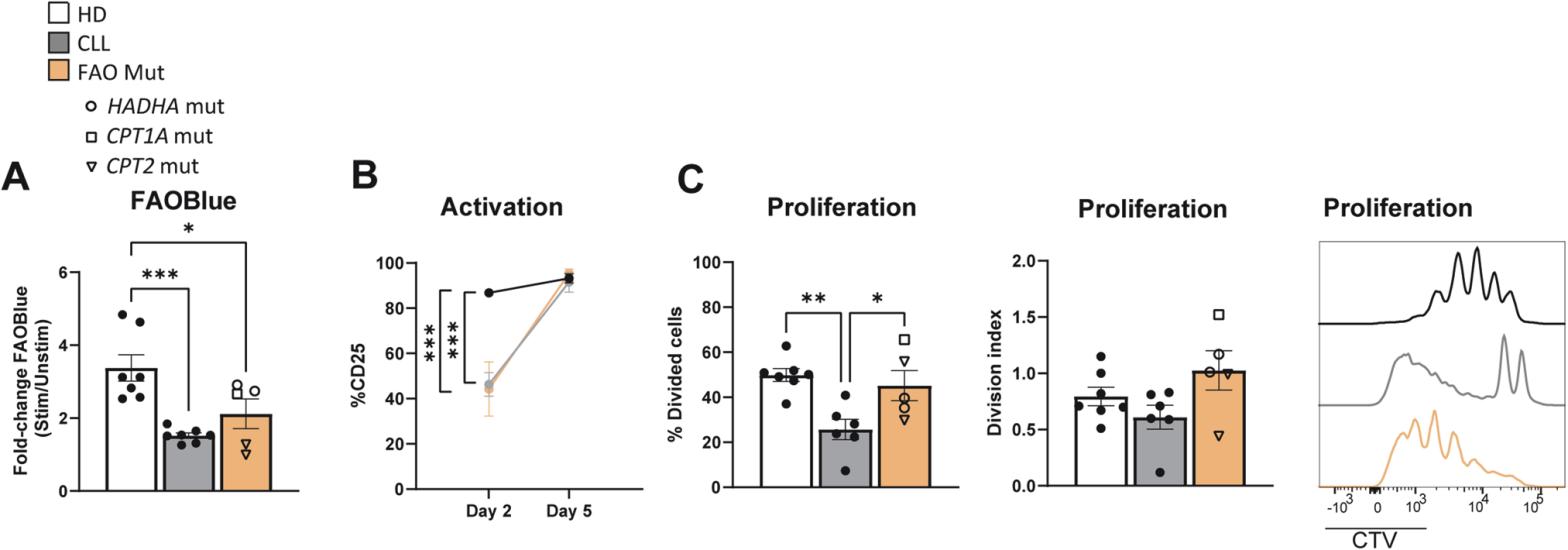
Defective FAO leads to delayed T-cell activation, but does not affect proliferation. PBMCs from HD, CLL patients and patients with genetic FAO defects (FAO Mut) were stimulated for 2 or 5 days with αCD3/αCD28 antibodies. Patients with *HADHA*, *CPT1A*, and *CPT2* mutations are indicated with circles, squares, and triangles, respectively. **A** FAO was evaluated in CD4+ T cells at day 2 by using FAOBlue, and fold change of intensity was calculated by dividing the fluorescence intensity of the stimulated samples by that of the matched unstimulated samples in all groups. **B** Expression of CD25 on CD4+ T cells was measured on days 2 and 5. **C** Proliferation was measured at day 5 and is represented by percentage of divided cells (left) and division index (middle). Representative CTV histograms are shown (right); HD (black), CLL (gray), and FAO Mut (orange). Data are presented as mean ± SEM and differences were analyzed with two-way repeated measures ANOVA with Tukey’s/Šidák’s multiple comparison test. *** = *p* < 0.001; ** = *p* < 0.01; * = *p* < 0.05

T cells from patients with FAO mutations (FAO Mut) exhibited reduced FAO upon αCD3/αCD28 stimulation, as expected,similar to the pattern observed in CLL patients (Fig. 6A, Supplementary Fig. 6A). Furthermore, FAO Mut T cells displayed a delayed increase in CD25 expression upon activation, similar to the phenomenon observed in CLL T cells [44] (Fig. 6B, Supplementary Fig. 6B). Notably, the proliferative capacity of T cells from FAO Mut patients was not impaired (Fig. 6C, Supplementary Fig. 6C).

Taken together, these data indicate that reduced FAO leads to delayed T-cell activation but does not affect proliferation. Therefore, we show for the first time that the diminished proliferation capacity of CLL T cells is likely not attributable to lower FAO, and may instead be due to low cholesterol levels.

Besides being a key building block for new membranes, cholesterol is an essential component of lipid rafts. These are ordered regions in the plasma membrane formed by self-aggregation of cholesterol and sphingolipids and recruit other lipids and proteins that are essential for the initiation and maintenance of T-cell signaling upon TCR ligation [54]. Subsequent actin polymerization and re-organization stabilizes the rafts and allows for sustained signaling, supporting T-cell activation and proliferation [54]. Consequently, the organization, size, and cholesterol content of lipid rafts are crucial for T-cell responses [54].

To identify whether cholesterol content is different between HD and CLL T cells, we compared absolute levels of CE from our lipidomics data set, which were found to be decreased (Supplementary Fig. 7A). CE and plasma membrane cholesterol have a common source, which is LDL-derived cholesterol [55]. Given that also LDLR expression was found decreased in CLL T cells (Fig. 1E), we investigated lipid raft formation in HD and CLL T cells.

**Fig. 7:**
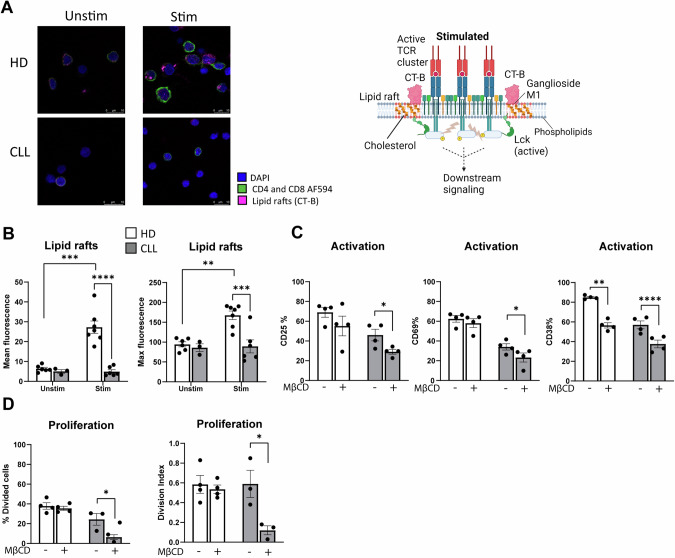
CLL T cells have altered membrane organization and disorganized lipid raft formation. **A** PBMCs from HD and CLL patients were stimulated with αCD3/αCD28 antibodies for 2 days. Immunofluorescence to assess the localization of lipid rafts on T cells was performed by staining samples with AF448-conjugated choleratoxin-B (CT-B), followed by incubation with AF594-conjugated antibodies against CD4 and CD8. DAPI was used as nuclear staining. Representative images of each experimental condition analyzed are shown (left). A schematic overview of lipid rafts localized around the T-cell receptor (TCR) in stimulated T cells and CT-B binding is provided (right). **B** CT-B intensity was quantified and plotted as total abundance (mean fluorescence, left) and clustering (maximal fluorescence, right) in HD and CLL T cells. Each dot represents the average value of CT-B fluorescence from all T cells in one field. **C** PBMCs from HD and CLL patients were either preincubated for 1 h with 2 mM methyl-β-cyclodextrin (MBCD), prior to a 2-day T-cell stimulation with αCD3/αCD28 antibodies in the continued presence of MBCD, or stimulated without MBCD present. Expression of CD25, CD69, and CD38 was measured on HD and CLL CD4+ T cells. **D** PBMCs from HD and CLL patients were stimulated with αCD3/αCD28 antibodies for 5 days in the same experimental conditions as in (**C**). Proliferation of CD4+ T cells is shown as percentage divided cells (left) and division index (right). Data are presented as mean ± SEM and differences were analyzed with two-way repeated measures ANOVA with Tukey’/Šidák’s s multiple comparison test (**B**) or with paired *t*-test (**C**, **D**). **** = *p* < 0.0001; *** = *p* < 0.001; ** = *p* < 0.01; * = *p* < 0.05

Using confocal microscopy, we examined HD and CLL T cells after 2 days of stimulation, employing CT-B, which binds to ganglioside M1, a glycolipid highly enriched in lipid rafts (schematically shown in Fig. 7A, right). Reduced CT-B signal was observed in CLL T cells (Fig. 7A, left), and quantification of these observations showed lower mean and maximal CT-B fluorescence, indicating decreased abundance and clustering of lipid rafts, respectively, in T cells from CLL patients compared to HD (Fig. 7B). This reduction was not due to differences in T-cell activation at time of measurement, as CLL PBMCs with similar activation levels compared to HD were specifically selected for this experiment. These findings demonstrate that CLL T cells not only have fewer lipid rafts per cell but also exhibit reduced clustering of these rafts. This observation is in line with our finding of intracellular lipid imbalances, particularly of cholesterol, sphingolipids, and phospholipids, in CLL cells.

To further investigate the importance of lipid rafts in T-cell function, we pre-treated T cells with MBCD to deplete membrane cholesterol and disrupt lipid rafts prior to T-cell stimulation [56]. Upon 2-day stimulation, confocal analyses were performed to analyze the structure of lipid rafts. Altered CT-B signal with decreased lipid rafts clustering was observed by confocal in activated T cells that had been exposed to MBCD (Supplementary Fig. 7B). In order to assess the effects of lipid raft disruption on T-cell function, activation and proliferation were measured in stimulated HD and CLL PBMCs upon MBCD exposure. Reduced expression of CD38, which is localized within lipid rafts [57], was found in both groups, while CD25 and CD69 were significantly decreased in CLL T cells, but not in HD (Fig. 7C, Supplementary Fig. 7C). In line with this, T-cell proliferation in HD was not affected by MBCD, while it was significantly dampened in CLL (Fig. 7D, Supplementary Fig. 7D), indicating again that compensatory mechanisms to preserve cholesterol homeostasis present in HD are lacking in CLL T cells.

In summary, our data reveal global dysregulation of lipid metabolism as a fundamental cause of T-cell dysfunction in CLL. These disturbances consist of a deficiency in cholesterol and phospholipids leading to diminished proliferative capacity and a disorganized membrane composition, which impairs lipid raft clustering. Additionally, altered lipid biosynthesis renders CLL T cells more susceptible to cholesterol depletion and, together with reduced FAO for energy production, further contributes to the lack of effective T-cell responses in CLL.

## Discussion

We present a comprehensive study of lipid metabolism in T cells from HD and CLL patients to dissect how lipids contribute to normal T-cell function, and how this is deregulated in CLL, as an example of a disease with tumor-induced T-cell dysfunction. Our findings demonstrate that cholesterol is crucial for T-cell proliferation, serving as a key component of new membranes and lipid rafts, while mitochondrial FAO supports T-cell activation and it is not essential for proliferation. In CLL, T-cell lipid metabolism is disturbed on multiple levels. Compared to age-matched HD, CLL T cells exhibit reduced uptake of exogenous cholesterol, an impaired ability to initiate de novo FA synthesis, decreased FAO, lower relative abundance of phospholipids and cholesterol, and increased TAG storage.

Cholesterol and lipid metabolism are crucial for T-cell function, influencing activation, proliferation, and signaling [23, 46]. Cholesterol maintains membrane properties essential for TCR clustering and IS formation [46]. Lipid metabolism integrates environmental cues and intracellular signals to regulate T-cell responses [46]. However, specific roles of cholesterol dynamics in T-cell function in general and particularly in cancers where T-cell function is affected, such as CLL, remain unclear.

Cellular lipid metabolism is orchestrated by distinct transcription factor families, primarily SREBP1/2, PPARα/γ, and LXR [24, 58]. An essential part of T-cell activation is the reciprocal regulation of these three families following TCR triggering, which specifically governs cell-cycle progression and proliferation [46, 48]. Our discovery of decreased activity of these transcriptional programs in CLL T cells demonstrates that lipid metabolism is globally dysregulated in these cells, which can explain their decreased proliferation capacity reported by previous studies [8, 11, 12], and corroborated here. It has been previously shown that reduced activity of SREBP2 leads to cholesterol deficiency due to diminished uptake and compromised synthesis [40]. A recent study by Yan et al. demonstrated that cholesterol deficiency drives tumor-infiltrating lymphocytes towards an exhausted and dysfunctional state in several solid tumor mouse models [59], in line with our findings in CLL. Specific lipid species act as natural ligands for these transcription factors, such as FA for PPARα/γ [60], and cholesterol and its biosynthesis intermediates for SREBP2 and LXR [58, 61–63]. Since both the expression of the main lipid transcription factors and the abundance of their ligands are reduced in CLL T cells, restoring lipid metabolism homeostasis would require a multilevel approach targeting various pathways simultaneously.

In order to pinpoint the exact lipid requirements for T-cell activation and proliferation, we analyzed T cells from patients with genetic mitochondrial FAO disorders and compared them with T cells from CLL patients. Our findings revealed that T-cell activation and proliferation require distinct branches of lipid metabolism. The delayed T-cell activation observed in patients with FAO defects coincides with the phenotype seen in CLL and evidence that FAO needs to be triggered upon TCR stimulation in order for T cells to accomplish full activation. Whilst FAO was shown to be essential for cell-cycle progression in different types of tumor cells [64], T cells from the patients with mutations in FAO analyzed in our study displayed a normal proliferation profile. Supporting this notion, studies have demonstrated that inhibiting FA synthesis -the opposite pathway to FAO- attenuates T-cell proliferation in HD T cells [26]. Thus, we conclude that the proliferation defects observed in CLL T cells are attributable to their lack of membrane lipids, including cholesterol and phospholipids, rather than reduced FAO itself.

Besides, a role for lipid competition imposed by malignant CLL cells also consuming lipids cannot be ruled out. As mentioned before, CLL cells have been shown to use lipids in the mitochondria [27] and express LPL, which is associated with poor prognosis [30]. It is important to note that in this study the effect of LPDS medium or lipid inhibitors on malignant CLL cells was not studied.

A crucial finding of our work is the presence of lipid accumulations in the cytoplasm of human T cells. This observation demonstrates that T cells possess the ability to sequester excess lipids as a protective mechanism against lipotoxicity, which has been reported before for adipocytes and other non-adipose cells [65]. Deregulated presence of free lipids can become toxic by causing ER stress and lead to activation of the unfolded protein response, ultimately harming T-cell function [65]. Our findings reveal that HD T cells accumulate lipids in PLIN2-positive structures, which fit the description of classical lipid droplets, whereas CLL T cells exhibit increased neutral lipid aggregates, not all of which are PLIN2-positive. Besides, our lipidomics data show that the main components of lipid droplets in healthy cells are CE, while TAG are the primary constituents of lipid accumulations in CLL T cells. The lipid composition of lipid droplets dictates which specific perilipin family member is expressed on their surface [66]. Therefore, lipid droplets in CLL T cells might express other perilipins, and consequently, distinct lipolysis enzymes may be required for their breakdown compared to HD T cells [66], which might also influence their subsequent capacity for FAO.

Besides the cholesterol accumulated in the cytoplasm in the form of CE, free cholesterol is required for membrane organization in lipid rafts, TCR clustering, and the formation of a functional IS. This has been previously shown in several cell lines and mouse primary T cells [67–69]. Reduced cholesterol uptake due to lower LDLR expression on mouse CD8+ tumor-infiltrating lymphocytes hampered their anti-tumor activity [70], which is in line with our findings in CLL. On the other hand, increasing free cholesterol in CD8+ T cells in a mouse tumor model was shown to improve T-cell effector function, proliferation and IS formation, ultimately leading to superior tumor and metastasis control [71]. Notably, LDLR directly interacts with the TCR complex, regulating its signaling and recycling, which favors effector function [70]. In Jurkat cells, major changes in lipid-lipid interactions, particularly involving cholesterol and re-organization of lipid rafts, were found to be crucial for effective IS formation [54, 68]. A well-known aspect of CLL T cells is that they exhibit impaired IS formation [10]. The observed disruption of lipid raft formation indicates a possible causal link between altered lipid membrane composition in CLL T cells and their disturbed IS formation, inferior T-cell effector function, and lack of success of autologous T cell-based therapies in this disease. The experiments we have performed depleting membrane-bound cholesterol, which led to further decreased activation and proliferation in CLL T cells, provide additional evidence of the important role of cholesterol scarcity in T-cell dysfunction in CLL.

In conclusion, our results demonstrate a faulty lipid regulation at the transcriptional level, which results in altered lipid composition and utilization in CLL T cells, contributing to T-cell dysfunction at multiple levels, the most important being impaired lipid raft formation and proliferation due to lack of cholesterol and phospholipids, and compromised bioenergetics by lower FAO. Thus, altered lipid metabolism constitutes an integral explanation for altered T-cell function in CLL. Strategies to increase lipid uptake and synthesis could rebalance lipid homeostasis and ameliorate this dysfunction. However, given the central role of lipids in T-cell function reported by this and previous studies, we think that strategies increasing cholesterol availability in CLL T cells are especially interesting to be applied during the ex vivo manufacturing of CAR-T cell products in this disease, and not as systemic treatment to patients. In this regard, two potential strategies could be proposed: (1) increasing the availability of intracellular cholesterol, leading to higher T-cell proliferation and thus CAR-T persistence, and (2) increasing FAO to boost T-cell activation and the acquisition of a memory phenotype. Manipulation of metabolic routes in CAR-T cells has been explored before [72, 73], and the first studies overexpressing lipid-related genes in T-cells have just been published [74]. Thus, we expect this to be a promising field in the future, and consider overexpression of specific transcription factors the most efficient approach to achieving global regulation of a specific pathway, while we acknowledge limitations on that with regards to transcription factor activity being situation- or ligand-dependent, which might be difficult to control. Strategies improving lipid homeostasis in T cells have the potential to improve depth and duration of CAR-T cell therapy responses by enhancing T-cell activation, proliferation, and ultimately anti-tumor responses, not only in CLL but also in other tumors with acquired T-cell dysfunction.

## Supplementary information

Supplementary figures and legends

Supplementary tables
