# Supplementary material for: Cholesterol homeostasis and lipid raft dynamics at the basis of tumor-induced immune dysfunction in chronic lymphocytic leukemia
Source: Cell Mol Immunol. 2025 Mar 4;22(5):485–500. doi: 10.1038/s41423-025-01262-1 (PMC12041523; doi:10.1038/s41423-025-01262-1)
Supplement: Supplementary file 1 — Supplementary figures and legends [file 41423_2025_1262_MOESM1_ESM.pdf]

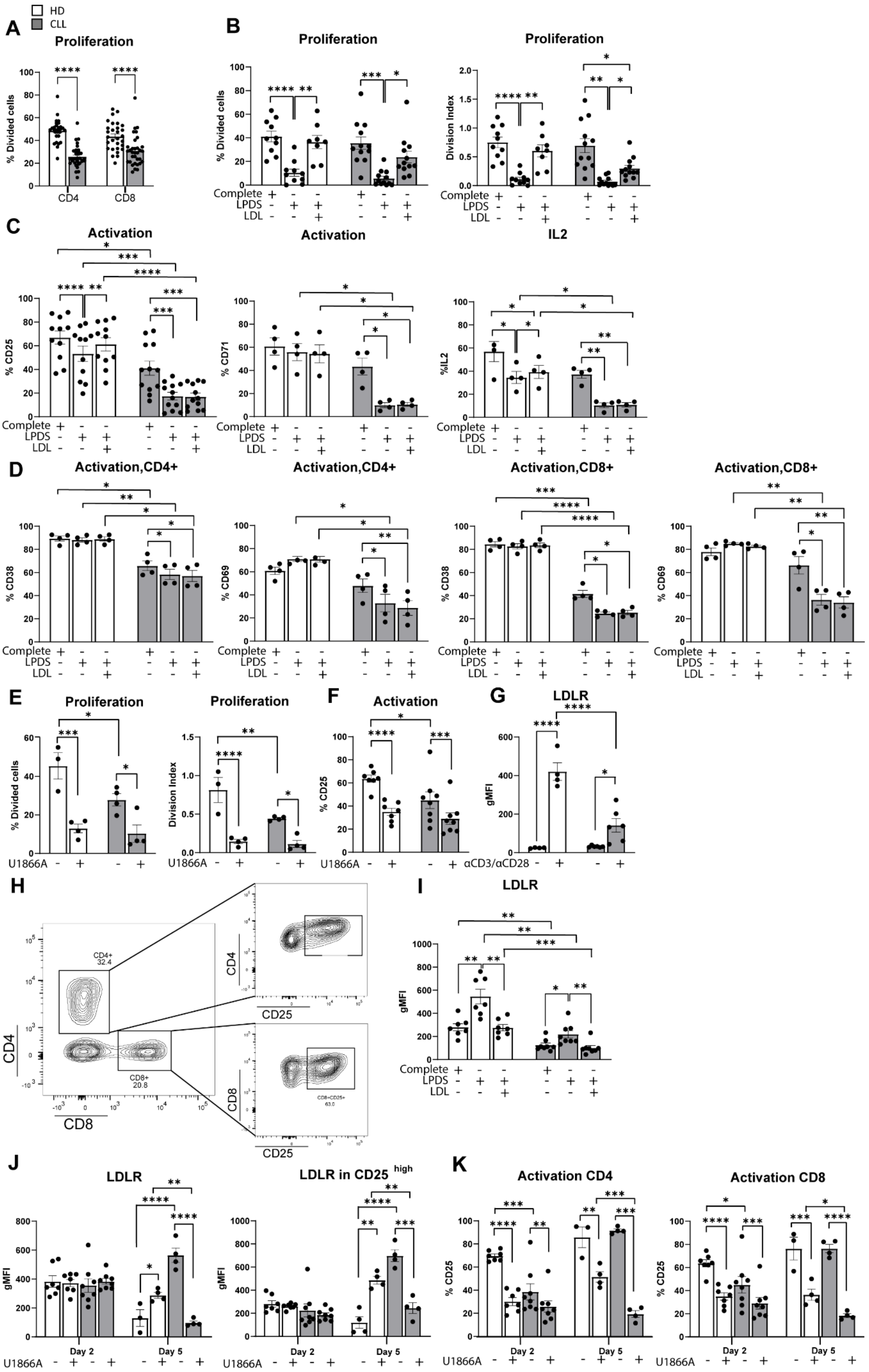

HD
CLL
A
Proliferation
% Divided cells
CD4
CD8
B
Proliferation
% Divided cells
Division Index
Complete
LPDS
LDL
C
Activation
% CD25
% CD71
IL2
%IL2
D
Activation,CD4+
Activation,CD8+
% CD38
% CD69
E
Proliferation
% Divided cells
Division Index
U1866A
F
Activation
% CD25
G
LDLR
gMFI
αCD3/αCD28
H
CD4+
32.4
CD8+
20.8
CD4
CD8
CD25
I
LDLR
gMFI
J
LDLR
LDLR in CD25 high
gMFI
Day 2
Day 5
K
Activation CD4
Activation CD8
% CD25

Suppl. Fig 1.

Extracellular cholesterol import is required for T-cell proliferation and is decreased in T cells from CLL patients

A) PBMCs from healthy donors (HD) and chronic lymphocytic leukemia (CLL) patients were stimulated with αCD3/αCD28 antibodies for 5 days. To assess proliferation, PBMCs were labelled with cell trace violet (CTV) before stimulation. CD4+ and CD8+ proliferation was measured after 5 days of stimulation and is expressed as percentage of divided cells. Data from all patients analyzed in this study has been included in this panel. **B)** Proliferation of CD8+ T cells was measured as percentage divided cells (left) and division index (right) after a 5-day stimulation with αCD3/αCD28 antibodies under complete serum conditions, lipid deprivation (lipoprotein deficient serum, LPDS) and LPDS supplemented with low-density lipoproteins (LDL). **C)** Expression of CD25, CD71 and IL2 (intracellularly, after 4 hours of Brefeldin treatment) was measured on CD8+ HD and CLL T cells after 2 days of stimulation in the same experimental conditions as in B. **D)** Expression of CD38 and CD69 was measured on CD4+ (left) and CD8+ (right) T cells after a 2-day stimulation in the same experimental conditions as in B. **E)** PBMCs from HD and CLL patients were stimulated with αCD3/αCD28 antibodies for 5 days in the presence or absence of the NPC1 inhibitor U18666A (10µM). Proliferation of CD8+ T cells is shown as percentage divided cells (left) and division index (right). **F)** Expression of CD25 was measured on HD and CLL T cells on day 2 in the same experimental conditions as in E. **G)** Expression of LDLR was measured by flow cytometry on CD8+ HD and CLL T cells after a 2-day stimulation. **H)** Example of gating strategy of CD4+ and CD8+ CD25^{high} T cells. **I)** Expression of LDLR was measured on CD8+ HD and CLL T cells after a 2-day stimulation in the same experimental conditions as in C. **J)** Expression of LDLR was measured after a 2-day stimulation in the presence or absence of the NPC1 inhibitor U18666A (10µM) on CD8+ T cells (left) and within and within CD8+ CD25^{high} T cells (right). **K)** Expression of CD25 on HD and CLL CD4+ (left) and CD8+ (right) T cells was measured on day 2 and day 5 upon stimulation with αCD3/αCD28 antibodies in the presence or absence of the NPC1 inhibitor U18666A (10µM). Data are presented as mean ± SEM and differences were analyzed with t-test (panel A) or two-way repeated measures ANOVA with Tukey's/Šidák's multiple comparison test (panels B, C, D, E, F, G, I, J and K). **** = p<0.0001; *** = p < 0.001; ** = p < 0.01; * = p < 0.05.

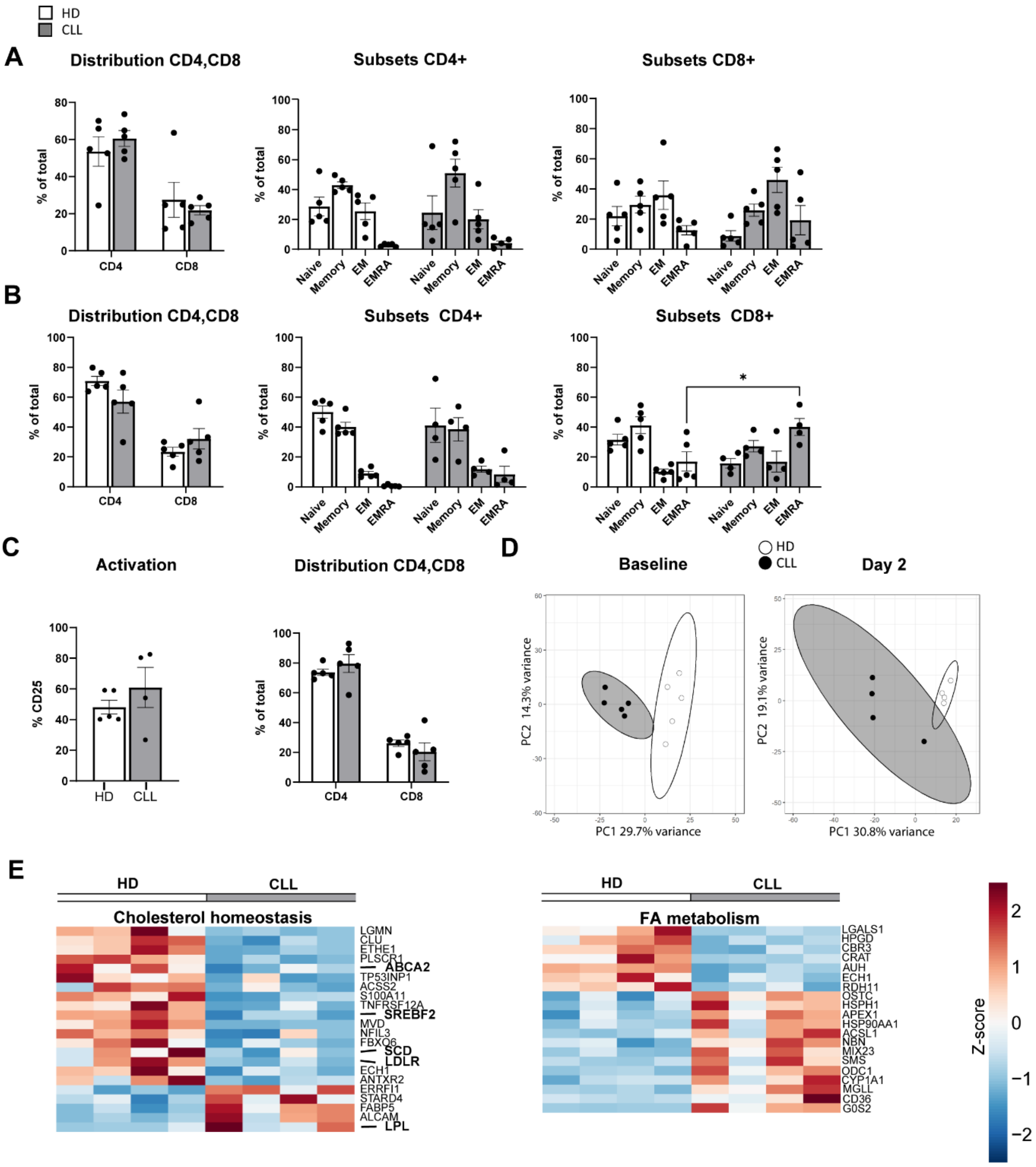

Suppl. Fig 2

Key transcriptional programs governing lipid metabolism are downregulated in CLL T cells

A-B) Before RNAseq experiments, T-cell subset distribution in PBMCs from HD and CLL patients was analyzed (left), and T-cell subsets were assessed in CD4+ (middle) and CD8+ (right), according to surface expression of CD45RA and CD27: naïve ($CD45RA^{+}CD27^{+}$), memory ($CD45RA^{-}CD27^{+}$), effector memory (EM, $CD45RA^{-}CD27^{-}$), and terminally differentiated effector memory (EMRA, $CD45RA^{+}CD27^{-}$). Samples were subsequently used for RNAseq at **A)** baseline, or **B)** after a 2-day stimulation with αCD3/αCD28. **C)** Expression of CD25 (left) and CD4+ and CD8+ distribution (right) were measured after a 2-day stimulation with αCD3/αCD28 antibodies in sorted T-cells prior to RNASeq. Data are presented as mean ± SEM and differences were analyzed with t-tests (panels A and B, left, and C) or two-way repeated measures ANOVA with Tukey's/Šidák's multiple comparison test (panels A and B, middle and right). * = $p < 0.05$. **D)** CD4+ and CD8+ T cells of HD and CLL patients were FACS-sorted and subjected

to RNA sequencing. Principal component analysis (PCA) was performed at baseline (left) or after a 2-day stimulation with αCD3/αCD28 antibodies (right). **E)** Heatmaps showing RNASeq results of differentially expressed genes (padj<0.05) involved in cholesterol homeostasis and FA metabolism in HD and CLL T cells after a 2-day stimulation with αCD3/αCD28.

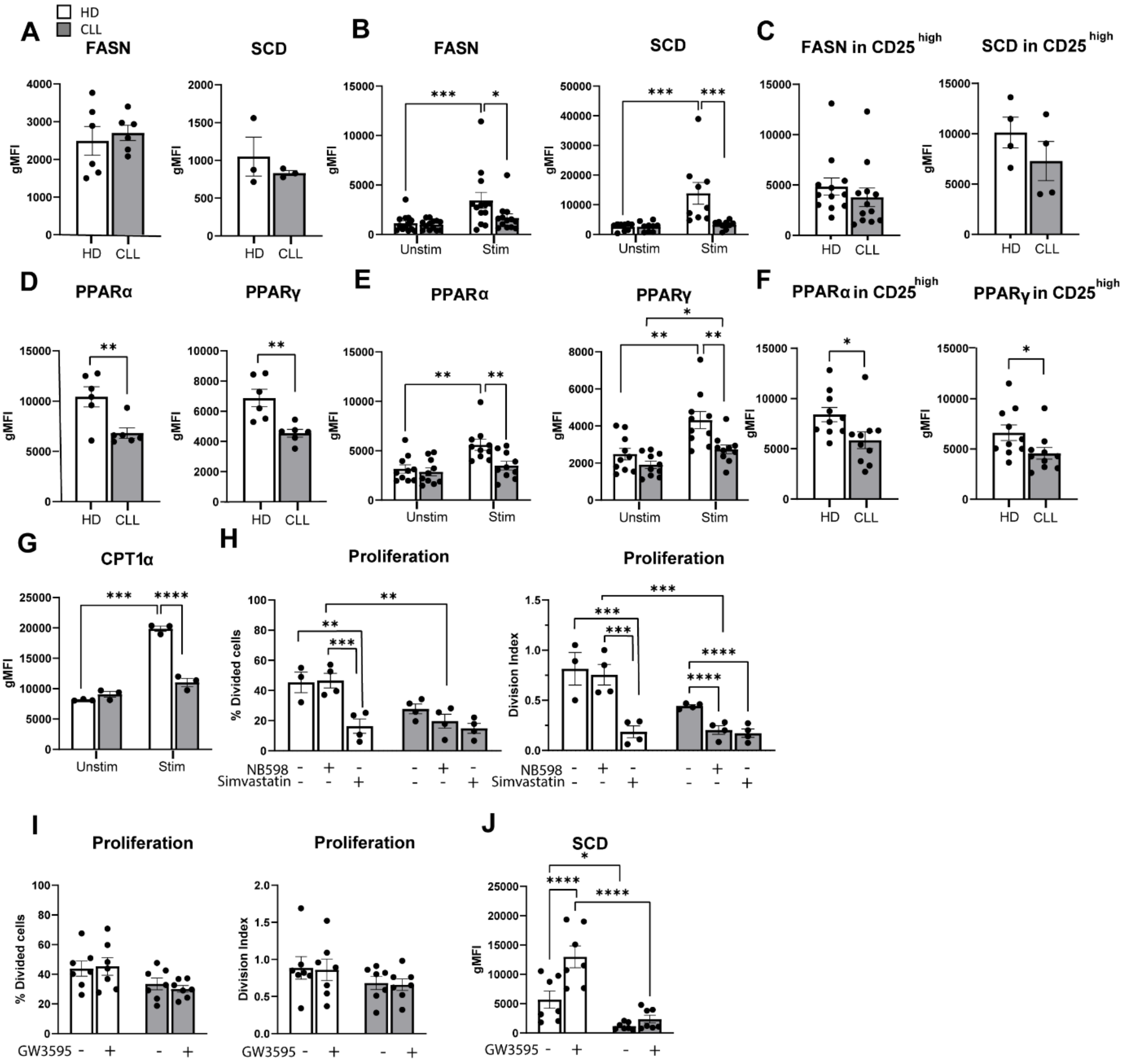

Suppl. Fig 3

Essential lipid metabolism proteins are downregulated in CLL T cells, which portray increased dependency on *de novo* cholesterol biogenesis

A) Levels of FASN and SCD were measured on CD8+ T cells from HD and patients at baseline, **B)** after a 2-day stimulation with αCD3/αCD28, and **C)** within CD8+ CD25high T cells. **D)** Expression of PPARα and PPARγ was measured on CD8+ T cells from HD and CLL patients at baseline, **E)** after a 2 day stimulation with αCD3/αCD28, and **F)** within CD8+ $CD25^{high}$ T cells by flow cytometry. **G)** Expression of CPT1α was measured on CD8+ T cells from HD and CLL patients after a 2-day stimulation with αCD3/αCD28. **H)** PBMCs from HD and CLL patients were labelled with CTV and stimulated with αCD3/αCD28 antibodies for 5 days in the presence or absence of the squalene inhibitor NB598 (10µM) or Simvastatin (10µM). Proliferation of CD8+ T cells is shown as percentage divided cells (left) and division index (right). **I)** PBMCs from HD and CLL patients were labelled with CTV and stimulated with αCD3/αCD28 antibodies for 5 days in the presence or absence of the LXR agonist GW3695 (1µM). Proliferation of CD8+ T cells is shown as percentage divided cells (left) and division index (right). **J)** Expression of SCD at day 2 was measured by flow cytometry on CD4+ T cells under the same experimental conditions as in I. Data are

presented as mean ± SEM and differences were analyzed with t-tests (panels A, C, D, and F) or two-way repeated measures ANOVA with Tukey's/Šidák's multiple comparison test (panels B, E, G, H, I and J). **** = $p<0.0001$; *** = $p < 0.001$; ** = $p < 0.01$; * = $p < 0.05$.

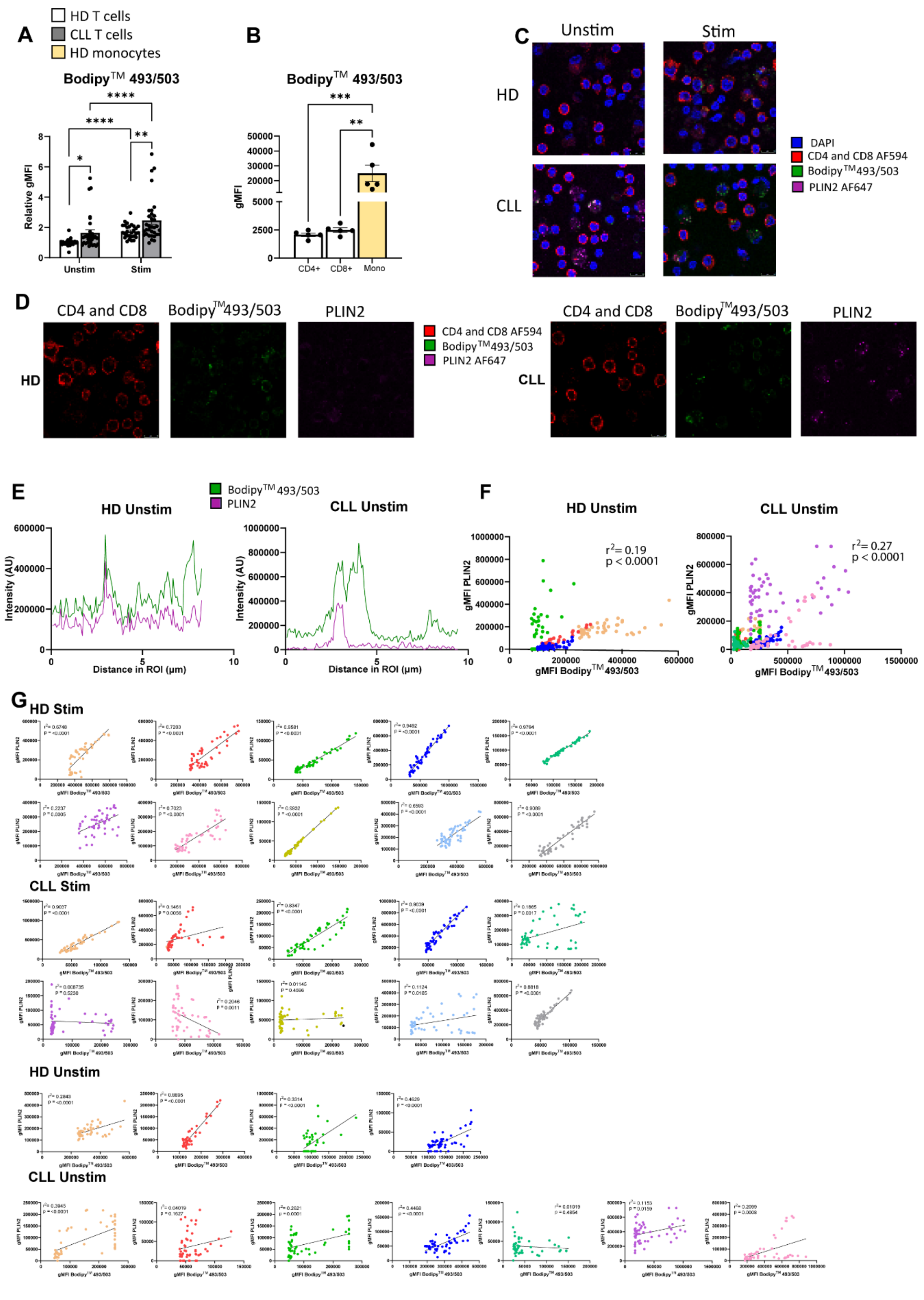

Suppl. Fig 4

The organization of lipids in the cytoplasm is different in T cells from CLL patients compared to HD

A) PBMCs from HD and CLL patients were stimulated with αCD3/αCD28 antibodies for 2 days. Neutral lipid accumulation was quantified using Bodipy™493/503 staining by flow cytometry on CD8+ T cells.

Raw gMFI values were normalized to the unstimulated HD samples in each independent experiment. **B)** Neutral lipid accumulation was quantified using Bodipy™493/503 staining at baseline in CD4+ and CD8+ T cells and monocytes (identified by FSC/SSC and CD4+ dim) from HD. **C)** Upon culturing in the same experimental conditions as in A, CD19 MACS depletion and was performed on the stimulated CLL PBMCs for T cell enrichment. Immunofluorescence was performed, first staining with Bodipy™493/503 and then with antibodies against CD4+ and CD8+ (both conjugated to AF549) and PLIN2 (unconjugated). A secondary antibody goat anti-mouse conjugated to AF647 was used to detect PLIN2, and DAPI was used as nuclear staining. Samples were imaged with confocal microscopy. Representative merged images of each experimental condition analyzed are shown. **D)** Single-channel images corresponding to the immunofluorescence images shown in Figure 4C. **E)** Upon culturing in the same experimental conditions as in A, immunofluorescence to assess the co-localization of Bodipy™493/503 with the lipid droplet marker PLIN2 was performed. Regions of interest (ROI) were manually selected based on Bodipy™493/503. Fluorescence intensity of Bodipy™493/503 and PLIN2 was quantified in ImageJ as gray values throughout each ROI and plotted in spatial plots to assess co-occurrence in a cross-sectional ROI. Representative spatial plots of fluorescence intensity from an unstimulated HD (left) and CLL (right) samples are shown. **F)** In each ROI, the 50 pixels with the highest Bodipy™493/503 fluorescence were selected. Intensity of Bodipy™493/503 and PLIN2 within the same pixel were plotted against each other and correlation was calculated. Data from all ROIs analyzed in unstimulated cells is shown. Every dot represents one pixel and every color corresponds to one ROI. **G)** Intensity of Bodipy™493/503 and PLIN2 within the same pixel. Every dot represents one pixel and every color and graph corresponds to one ROI. The coefficients of correlation and p-values shown were used to identify the cells with a significant positive correlation of Bodipy™493/503 and PLIN2 ($p<0.05$). Data are presented as mean ± SEM (panels A and B) and differences were analyzed with two-way repeated measures ANOVA with Tukey's/Šidák's multiple comparison test (panel A), t-test (panel B), or linear regression analysis (panels E, F and G). *** = $p < 0.001$; ** = $p < 0.01$; * = $p < 0.05$.

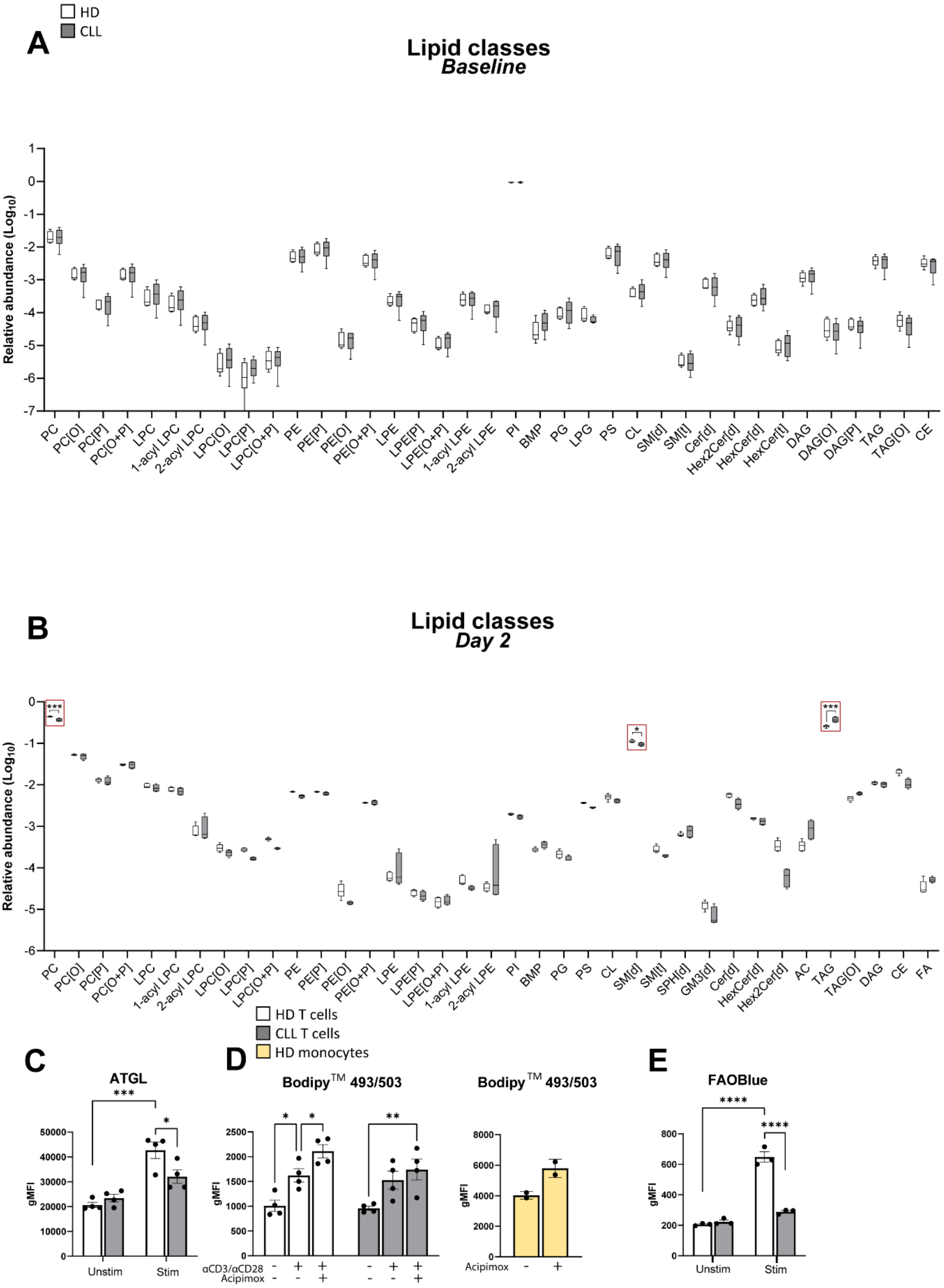

<u>Suppl. Fig 5</u>

The lipidome of CLL T cells is characterized by low cholesterol and phospholipids, and accumulation of triglycerides, compared to healthy T cells

CD4+ and CD8+ T cells of HD and CLL patients were FACS-sorted and subjected to liquid chromatography-mass spectrometry (LC-MS)-based lipidomics. Relative abundance of all lipid classes identified **A)** at baseline or **B)** after 2-day stimulation with αCD3/αCD28 antibodies is shown. Abundance of each lipid molecule was normalized by internal standards and protein amount (A) or by total lipidome pool (B). Abundance of the different lipid classes was calculated relative to the total lipids sum. Main classes of interest are highlighted with red squares. **C)** PBMCs from HD and CLL patients were stimulated for 2 days with αCD3/αCD28 antibodies and ATGL protein levels were measured. **D)** Bodipy^{TM}493/503 fluorescence was measured on HD and CLL T cells (left) and HD monocytes (identified by FSC/SSC and CD4+ dim) (right) stimulated for 2 days with αCD3/αCD28 antibodies in the presence or absence of the ATGL inhibitor Acipimox (1mM). **E)** Upon the same experimental conditions as in C, fatty acid oxidation (FAO) was assessed by flow cytometry on CD8+ T cells by FAOBlue. Data are presented as mean ± SEM and differences were analyzed with two-way repeated measures ANOVA with Tukey’s/Šídák's multiple comparisons test. **** = p<0.0001; *** = p < 0.001; ** = p < 0.01; * = p < 0.05.

PC: phosphatidylcholine, PC[O]: alkylphosphatidylcholine, PC[P]: alkenylphosphatidylcholine, PC[O+P]: alkyl/alkenylphosphatidylcholine, LPC: lysophosphatidylcholine, LPC[O]:alkyllysophosphatidylcholine, LPC[P]:alkenyllysophosphatidylcholine, LPC[O+P]: alkyl/alkenyllysophosphatidylcholine, PE: phosphatidylethanolamine, PE[P]:alkenylphosphatidylethanolamine, PE[O]:alkylphosphatidylethanolamine, PE[O+P]: alkyl/alkenylphosphatidylethanolamine, LPE: lysophosphatidylethanolamine, LPE[P]:alkyllysophosphatidylethanolamine, LPE[O+P]: alkyl/alkenyllysophosphatidylethanolamine, PI: phosphatidylinostiol, BMP: bis(monoacylglycero)phosphate, PG: phosphatidyglycerol, PS: phosphatidylserine, CL: cardiolipin, SM[d]: sphingomyeline, SM[t]: hydroxysphingomyeline, SPH[d]: sphingosine, GM3: monosialdihexosylganglioside, Cer[d]: ceramide, HexCer[d]: hexosylceramide, Hex2Cer[d]: dihexosylceramide AC: acylcarnitine, TAG: triacylglycerol, TAG[O]:alkyltriacylglycerol, DAG: diacylglycerol, CE: cholesterol ester, FA: fatty acid

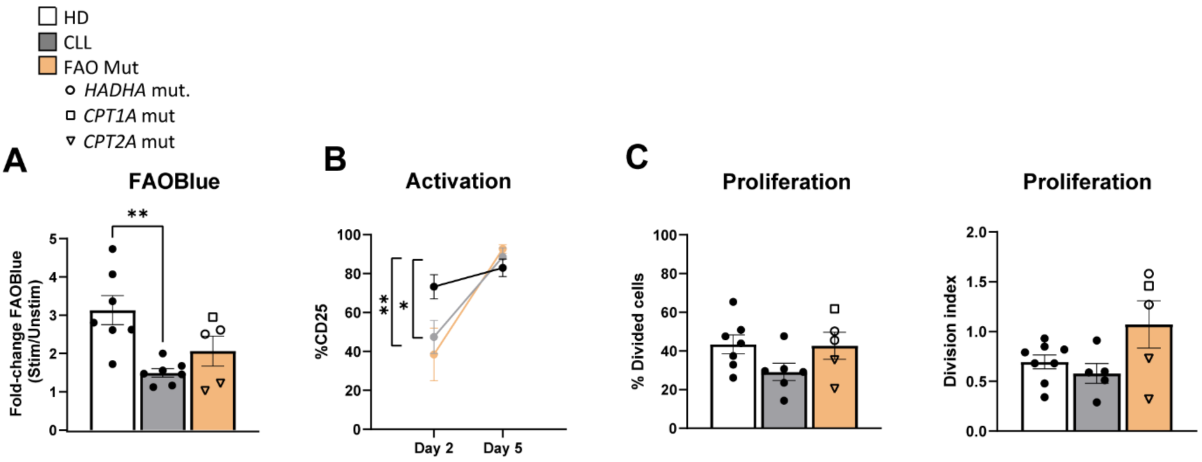

Suppl. Fig 6

Defective FAO leads to delayed T-cell activation, but does not affect proliferation

PBMCs from HD, CLL patients and patients with genetic FAO defects (FAO Mut) were stimulated for 2 or 5 days with αCD3/αCD28 antibodies. Patients with *HADHA*, *CPT1A* and *CPT2* mutations are indicated with circles, squares and triangles, respectively. **A)** FAO was evaluated in CD8+ T cells at day 2 by using FAOBlue, and fold-change of intensity was calculated by dividing the fluorescent intensity of the stimulated samples by that of the matched unstimulated samples in all groups. **B)** Expression of CD25 on CD8+ T cells was measured at days 2 and 5. **C)** Proliferation of CD8+ T cells was measured at day 5 and is represented by percentage divided cells (left) and division index (right). Data are presented as mean ± SEM and differences were analyzed with two-way repeated measures ANOVA with Tukey's/Šidák's multiple comparison test. **** = $p<0.0001$; *** = $p < 0.001$; ** = $p < 0.01$; * = $p < 0.05$.

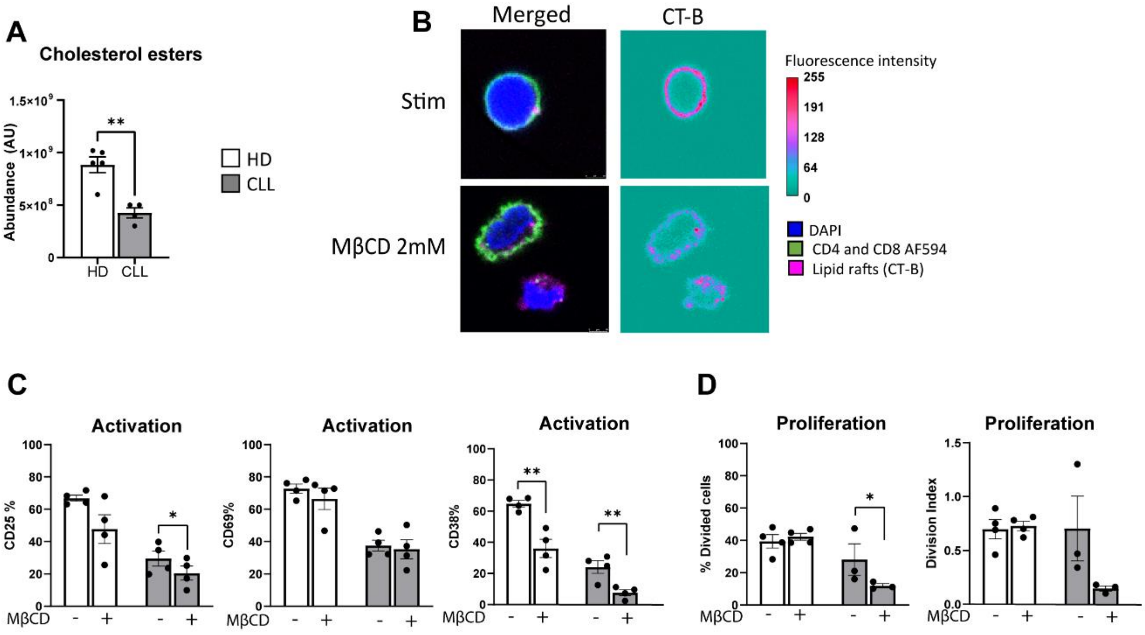

<u>Suppl. Fig 7</u>

CLL T cells have altered membrane organizations and disorganized lipid raft formation

PBMCs from HD and CLL patients were stimulated for 2 days with αCD3/αCD28 antibodies. **A)** CD4+ and CD8+ T cells of HD and CLL patients were FACS-sorted and subjected to liquid chromatography-mass spectrometry (LC-MS)-based lipidomics after a 2-day stimulation with αCD3/αCD28 antibodies. Absolute abundance of cholesterol esters is plotted. **B)** PBMCs from HD were either pre-incubated for one hour with 2 mM methyl-β-cyclodextrin (MBCD), prior to a 2-day T-cell stimulation with αCD3/αCD28 antibodies in the continued presence of MBCD, or cultured without MBCD present. Immunofluorescence was performed, first staining with CT-B AF488 and then with antibodies against CD4+ and CD8+ (both conjugated to AF549). DAPI was used as nuclear staining. Representative merged images and single-channel CT-B images are shown. **C)** Upon culturing in the same experimental conditions as in B, expression of CD25, CD69 and CD38 was measured on HD and CLL CD8+ T cells. **D)** PBMCs from HD and CLL patients were stimulated with αCD3/αCD28 antibodies for 5 days in the same experimental conditions as in B. Proliferation of CD8+ T cells is shown as percentage divided cells (left) and division index (right). Data are presented as mean ± SEM and differences were analyzed with unpaired t-test (panel A) or paired t-test (panels C and D).** = $p < 0.01$; * = $p < 0.05$.
